# Sequence effect on the self-assembly of discrete amphiphilic co-oligomers with fluorene-azobenzene semirigid backbones†

**DOI:** 10.1039/d3ra04205g

**Published:** 2023-08-11

**Authors:** Liandong Ye, Min Liu, Xiao Wang, Zhihong Yu, Zhihao Huang, Nianchen Zhou, Zhengbiao Zhang, Xiulin Zhu

**Affiliations:** a Suzhou Key Laboratory of Macromolecular Design and Precision Synthesis, Jiangsu Key Laboratory of Advanced Functional Polymer Design and Application, State and Local Joint Engineering Laboratory for Novel Functional Polymeric Materials, College of Chemistry, Chemical Engineering and Materials Science, Soochow University Suzhou 215123 China nczhou@suda.edu.cn zhangzhengbiao@suda.edu.cn

## Abstract

Sequences can have a dramatic impact on the unique properties and self-assembly in natural macromolecules, which has received increasing interest. Herein, we report a series of discrete amphiphilic co-oligomers with the same composition but different building blocks in a semirigid backbone. These sequence-defined oligomers possess two primary amine groups on the side chain of the azobenzene building block, and hence, they become amphipathic due to quaternization of the amine groups when protonated in acidic aqueous solution. These oligomer isomers assembled into different nanoparticles, including nanofibers, hollow vesicles and spherical micellar complexes, in a THF/water/HCl mixture under the same conditions. UV-vis absorption spectra, differential scanning calorimetry (DSC) and X-ray scattering (XRD) experiments combined with theoretical calculations reveal that the sequence-controlled co-oligomers induce different molecular packing conformations and arrangement modes of building blocks in self-assembly. Furthermore, these self-assembled nanoparticles demonstrate photoresponsive morphological transformation and fluorescence emission under UV light irradiation due to *trans*-to-*cis* photoisomerization of azobenzene. This work demonstrates that customizing functional nanoparticles can be achieved by controlling the sequence structure in synthetic co-oligomers.

## Abstract 2

Three discrete amphiphilic co-oligomers isomers with different sequences of building blocks in semirigid backbones assembled into different nanoparticles including nanofibers, hollow vesicles and spherical micellar complexes.

## Graphical abstract

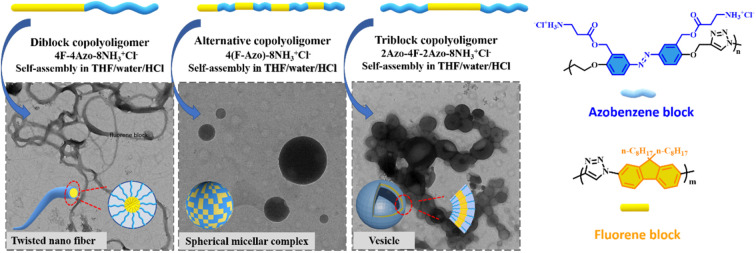

## Introduction

Natural macromolecules, such as DNA or protein, possess a precisely defined composition, sequence, and three-dimensional structure. Very slight variations in simple building blocks can make a large difference in properties and performances. Over the past years, the synthesis of sequence-controlled polymers has aroused wide attention to imitate biomacromolecules for developing artificial materials. With the tremendous progress in synthesis technology, chemists have been able to successfully fabricate synthetic macromolecules with well-defined compositions and sequence structures. Some pioneering groups have devoted themselves to a thorough and continuing study and made great contributions, although it has been a challenging task in recent decades.^1–10^ These new polymer materials have displayed interesting properties comparable to, or even surpassing those of, natural biopolymers in applications ranging from data storage to biotechnology.^11–15^

The primary driving force of all living and nonliving individuals in nature comes from assembly. Solution self-assembly of amphiphilic block copolymers is a particularly important class that provides a “bottom-up” simple and efficient preparation of supermolecule nanoparticles. Morphological control *via* self-assembly has attracted wide attention as a promising approach to create versatile nanomaterials with many potential applications in biomedical materials, microelectronics, photoelectric materials, catalysts and other fields.^16–31^ Rigid amphiphilic block copolymers containing conjugated groups in a backbone usually allow molecular chains to assemble along the 1D/2D direction toward smart nanoparticles due to interchain π–π stacking.^24,28–31^ Azobenzene (Azo) is a conjugated group and a typical photoresponsive group. The introduction of Azo units into polymer nanoparticles can endow their photostimulating response performance because of the *trans*–*cis* isomerization of azobenzene.^32–40^ In addition, the self-assembly of ionic block copolymers is intriguing for the preparation of smart nanoparticles because of their highly tunable structure and various electrostatic properties in response to external stimuli, such as changes in solvent pH or ionic strength.^1,41–44^

Well-defined oligomers of relatively low molecular weight are effective tools for achieving a deeper understanding of structure-controlled performance due to the difficulties in structural analysis and mechanistic insight of high molecular weight polymers thus far. Most investigations have demonstrated that small changes in repeating segments in synthetic polymers might induce substantial changes in the macroscopic physical properties and macromolecular self-assembly. Well-defined structures play a critical role in enabling the unique properties and functions of polymer materials, as described above. Inspired by this, our group has concentrated on this subject and made tremendous efforts. Previously, we investigated the solution self-assembly of a series of discrete amphiphilic polymers/oligomers containing azobenzene in the main chain. The semirigid backbone of discrete polymers/oligomers and precise chemical structures can induce cooperative supramolecular interactions such as π–π stacking of azobenzene and hydrogen bonding interactions within the aggregates, and hence, various smart 1D/2D nanostructures, including nanofibers, nanobelts and nanosheets, were obtained by self-assembly.^31,33^ In our current work, we further investigated the self-assembly of sequence-controlled discrete amphiphilic oligomers with azobenzene and fluorine building blocks in a semirigid main chain. The investigation shows the remarkable impact of the monomer sequence in these co-oligomers on one of the self-assembled properties in solution, *i.e.*, the morphology of the assembled aggregates.

**Scheme 1:**
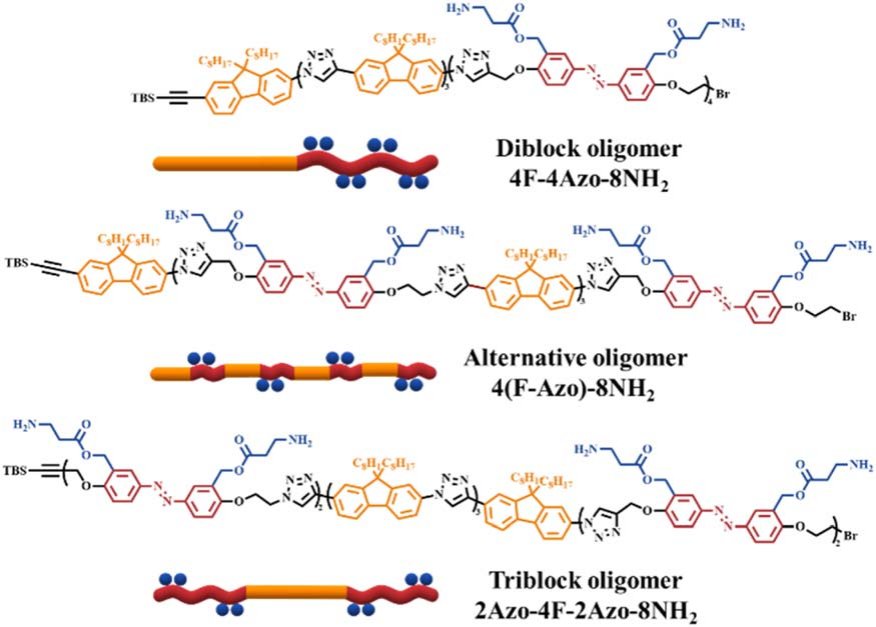
Molecular structures and schematic illustration of three sequence-defined isomeric co-oligomers of 4F-4Azo-8NH_2_, 2Azo-4F-2Azo-8NH_2_, 4(F-Azo)-8NH_2_.

In this work, two types of monomers containing azobenzene units and fluorene units were reasonably designed and synthesized to achieve fluorene-azo oligomers with precise chemical structures and different azobenzene sequences *via* iterative stepwise growth combined with a protection-deprotection strategy using an efficient copper(i)-catalyzed alkyne–azide cycloaddition (CuAAC) reaction. Three oligomer isomers named 4F-4Azo-8Boc-8NH_2_, 2Azo-4F-2Azo-8NH_2_, and 4(F-Azo)-8NH_2,_ which possess eight primary amine groups on the side chain of the azobenzene building block, are hydrophobic and become hydrophilic quaternary ammonium salts when protonated in acidic aqueous solution. The molecular structures of the oligomers are shown in Scheme 1, and their synthetic details are shown in the synthetic part above and in the ESI.† The self-assemblies of co-oligomers were conducted in a THF/water/HCl mixture, showing observations of nanofibers, hollow vesicles and spherical micellar complexes obtained by the three isomers under the same assembly conditions. The morphology and size of self-assembled aggregates was confirmed by TEM, SEM, AFM and DLS measurements. UV-vis absorption spectra, calorimetry (DSC) and X-ray scattering (XRD) were used to investigate the molecular stacking structure of the oligomers. Combined with theoretical calculations, the results show that sequence-controlled self-assembly induces distinguished molecular packing conformations in the aggregates of co-ligomer isomerism. In addition, the oligomer nanoparticles demonstrate photoresponsive morphological transformation and fluorescence emission under UV light irradiation due to *trans*-to-*cis* photoisomerization of azobenzene. This work demonstrates that customizing the multilevel supramolecular assembly structure and obtaining functional nanoparticles can be achieved by controlling the sequence structure of synthetic macromolecules.

## Experimental design

### Materials

*N*,*N*,*N*′,*N*′′,*N*′′-Pentamethyldiethylenetriamine (PMDETA; 98%; J&K) was dried with 4 Å molecular sieves and distilled three times under reduced pressure. Copper(i) bromide (CuBr, chemically pure; Shanghai Chemical Reagent, Shanghai, China) was first washed with excess acetic acid, followed by ethanol and ether, and then dried in a vacuum. Oxone (95%; Energy-chemical), 2-hydroxy-5-nitrobenzyl alcohol (98%; TCI), 2-hydroxybenzyl alcohol (98%; Macklin), bis(triphenylphosphine) palladium(ii) chloride (98%; Aladdin), *tert*-butyl(ethynyl)dimethylsilane (95%; Aladdin), 2-bromofluorene (99%; Aladdin), 1-bromooctane (99%; Energy-chemical), sodium azide (NaN_3_, 99.5%; Aldrich), tetrabutylammonium fluoride (TBAF; 1.0 mol L^−1^ in tetrahydrofuran (THF), Energy-chemical), and triethylamine (TEA; Analytical reagent) were used as received. All solvents in this article were dried with activated molecular sieves before use. Unless otherwise specified, all chemicals were purchased from Shanghai Chemical Reagent Co. Ltd, Shanghai, China and used as received.

### Characterizations

^1^H nuclear magnetic resonance (NMR) spectra were recorded on a Bruker Nuclear Magnetic Resonance instrument (300 MHz) using CDCl_3_ or DMSO-d_6_ as the solvent and tetramethylsilane (TMS) as the internal standard at room temperature (25 °C). The number-average molecular weight (*M*_n_) and polydispersity index (PDI = *M*_w_/*M*_n_) of the oligomers were measured using a TOSOH HLC-8320 size exclusion chromatography system equipped with refractive index and UV detectors and using two TSKgel Super Mutipore HZ-N (4.6 × 150 mm, 3 μm bead size) columns arranged in series for molecular weights ranging from 700–2 × 105 g mol^−1^. Tetrahydrofuran (THF) was used as the eluent at a flow rate of 0.35 mL min^−1^ at 40 °C. Data acquisition was performed using EcoSEC software, and molecular weights were calculated with polymethyl methacrylate (PS) standards. Matrix-assisted laser desorption/ionization time of flight mass spectrometry (MALDI-TOF-MS) was performed with a 355 nm nitrogen laser. The matrix compound 2-[3-(4-*tert*-butylphenyl)-2-methyl-2-propenylidene]-malononitrile (DCTB, Aldrich, >98%) was prepared (at a concentration of *ca.* 20 mg mL^−1^ in chloroform), and all samples were dissolved in CHCl_3_ (at a concentration of *ca.* 10 mg mL^−1^). Sodium trifluoroacetate prepared in ethanol at a concentration of 10 mg mL^−1^ was used as a cationizing agent. The matrix and cationizing salt solutions were mixed in a ratio of 10/1 (v/v). This mixture solution (0.5 μL) was placed on a metal sample plate. Fourier transform infrared (FT-IR) spectra were recorded on a Bruker TENSOR-27 FT-IR spectrometer. Differential scanning calorimetry (DSC) was conducted with a TA Instrument DSC2010 under the protection of nitrogen with a heating/cooling rate of 10 °C min^−1^. X-ray scattering (XRD) experiments were carried out on an X-ray scattering instrument equipped with line collimation and a 2000 W sealed-tube X-ray generator (Cu-Kα, *λ* = 0.154 nm). The data were collected using a Ni-filtered Cu-target tube at room temperature in the 2*θ* range from 3° to 90°. UV-vis absorption spectra of the oligomers were recorded on a Shimadzu UV-2600 spectrophotometer at a concentration of 2.0 × 10^−2^ mg mL^−1^ in chloroform. The light source used for the optical response test was a diode pumped solid-state laser (DPSSL), purchased from LASEVER INC. The fluorescence emission spectra of the assembled solutions of oligomers in THF/water/HCl were obtained on a HITACHI F-4600 fluorescence spectrophotometer. Transmission electron microscopy (TEM) was recorded on a HITACHI HT7700 at a 120 kV accelerating voltage. Scanning electron microscopy (SEM) images were recorded on a HITACHI S-4700 at a 15 kV accelerating voltage. The surface morphology was measured by atomic force microscopy (AFM) in tapping mode (Veeco Instruments Inc., Nanoscope IV). The hydrodynamic diameter (*D*_h_) was determined by dynamic laser scattering (DLS) using a Brookhaven NanoBrook 90Plus PALS instrument at 25 °C with a scattering angle of 90°. Molecular geometries were optimized using the DFT method of a three-parameter Becke-style hybrid functional (B3LYP) with the eople basis set 6-31+G (d,p). All theoretical calculations were performed using the GAUSSIAN 2009 package.

## Experimental procedures

### Synthesis of both monomers of fluorene TBS-F-NH_2_ and azobenzene TBS-Azo-Br-2Boc

The synthetic routes of TBS-F-NH_2_ and TBS-Azo-Br-2Boc are shown in Scheme S1,† and the synthetic process was conducted according to a previous study (ref. S1 and S2).† Their structural characterization is shown in the ESI (Fig. S1 and S2).†

### Synthesis of fluorene blocks TBS-2F-NH_2_ and TBS-4F-NH_2_

The synthesis process of TBS-2F-NH_2_ and TBS-4F-NH_2_ was conducted according to a previous study (ref. S1).† The synthetic routes and structural characterization are shown in the ESI (Scheme S2 and Fig. S3).†

### Synthesis of the azobenzene blocks TBS-2Azo-Br-4Boc and TBS-4Azo-Br-8Boc

The synthesis routes of TBS-2Azo-Br-4Boc and TBS-4Azo-Br-8Boc are shown in Schemes S2 and S5.† Structural characterization details of TBS-2Azo-Br-4Boc and TBS-4Azo-Br-8Boc are shown in Fig. S4.†

### Synthesis process of TBS-2Azo-Br-4Boc

Synthesis of TBS-Azo-N_3_-2Boc: TBS-Azo-Br-2Boc (66.92 mg, 1.03 mmol) and NaN_3_ (9.95 mg, 0.153 mmol) were dissolved in 5 mL DMF, and the reaction was stirred at 80 °C for 12 h. After the mixture cooled to room temperature, it was extracted with ethyl acetate and washed with a saturated NaCl solution three times, and the organic phase was collected and dried to obtain the product TBS-Azo-N_3_-2Boc as an orange-yellow goo (63.46 mg) with a yield of approximately 99.0%.

Synthesis process of alkyne-Azo-Br-2Boc: TBS-Azo-Br-2Boc (200 mg, 0.23 mmol) was dissolved in 10 mL tetrahydrofuran (THF), tetrabutylammonium fluoride (TBAF) in THF (300 μL, 1.14 mmol) was added into the mixture and stirred for 0.5 h at room temperature. After completion of the reaction monitored by TLC, the THF was removed by rotary evaporation and extracted with ethyl acetate. The organic phase was washed three times with a saturated NaCl solution, dried over Na_2_SO_4_ and concentrated *in vacuo* to obtain alkyne-Azo-Br-2Boc as a brown solid (171.29 mg, 98.5%).

In a 10 mL Schlenk tube, TBS-Azo-N_3_-2Boc (85.69 mg, 0.11 mmol) and alkyne-Azo-Br-2Boc (117.00 mg, 0.15 mmol) were dissolved in 5 mL of anhydrous THF, and then PMDETA (26.69 mg, 0.15 mmol) and CuBr (14.63 mg, 0.11 mmol) were added. The Schlenk tube was filled with argon three times to deoxygenate and reacted for 48 h at room temperature. After the reaction, the THF was removed by rotary evaporation, and the organic phase was extracted with ethyl acetate, washed three times with saturated NaCl solution, dried over Na_2_SO_4_ and concentrated *in vacuo*. The crude product was purified by column chromatography (silica gel, eluent: petroleum ether/ethyl acetate = 1/4, v/v) to yield an orange-red solid (150.56 mg, 92.1%). ^1^H NMR (300 MHz, CDCl_3_), *δ* (TMS, ppm): 7.94 (s, 1H, =CH–N–), 7.81 (tt, 8H, ArH), 7.09 (dd, 2H, ArH), 6.86 (d, 2H, ArH), 5.30–5.11 (m, 8H, –CH_2_–OH), 5.07 (s, 4H, –C–CH_2_–O), 4.75 (d, 4H, –CH_2_), 4.43–4.31 (t, 4H, –CH_2_), 3.37–3.25 (m, 8H, –CH_2_–NH), 2.51 (qd, 8H, –COO–CH_2_–), 1.31 (d, 36H, –CH_3_), 0.79 (s, 9H, –Si–(CH_3_)_3_), 0.08 (s, 6H, –Si–CH_3_).

### Synthesis of azobenzene blocks TBS-4Azo-Br-8Boc

The synthesis of TBS-4Azo-8Boc-Br-8Boc was performed by the “CuAAC” reaction of TBS-2Azo-N_3_-4Boc and alkyne-2Azo-Br-4Boc, which was the same as the synthesis method of TBS-2Azo-Br-4Boc above. ^1^H NMR (300 MHz, CDCl_3_), *δ* (TMS, ppm): 8.01 (s, 3H, =CH–N–), 7.95–7.68 (m, 16H, ArH), 7.24–7.04 (m, 4H, ArH), 6.96 (t, 4H, ArH), 5.52–5.04 (m, 16H, –CH_2_–OH), 4.96–4.80 (m, 8H, –C–CH_2_–O), 4.61–4.23 (m, 8H, –CH_2_), 4.21–3.57 (m, 8H, –CH_2_), 3.41 (s, 16H, –CH_2_–NH), 2.61 (q, 16H, –COO–CH_2_–), 1.41 (d, 72H, –CH_3_), 0.89 (s, 9H, –Si–(CH_3_)_3_), 0.09 (d, 6H, –Si–CH_3_).

### Synthesis of discrete co-oligomer precursors 4F-4Azo-8Boc, 2Azo-4F-2Azo-8Boc and 4(F-Azo)-8Boc

#### Synthesis of discrete 4F-4Azo-8Boc (diblock co-oligomer precursor)

In a 50 mL Schlenk tube, TBS-4F-N_3_ (322.00 mg, 0.17 mmol) and yne-4Azo-8Boc-Br-8Boc (162.50 mg, 0.06 mmol) were dissolved in anhydrous THF, and then PMDETA (28.65 mg, 0.17 mmol) and CuBr (15.78 mg, 0.11 mmol) were added. The mixture was degassed using three freeze–pump–thaw cycles and stirred for 48 h at room temperature. After the reaction, the reaction mixture was concentrated by rotary evaporation to remove THF and then extracted with ethyl acetate. The organic phase was washed three times with a saturated NaCl aqueous solution, dried over Na_2_SO_4_ and concentrated *in vacuo*. The crude product was purified by column chromatography (silica gel, eluent: petroleum ether/tetrahydrofuran = 1/2, v/v) to yield an orange-red solid (215.32 mg, 79.8%). ^1^H NMR (300 MHz, CDCl_3_), *δ* (TMS, ppm): 8.52–8.19 (m, 7H, =CH–N–), 8.16–6.97 (m, 48H, ArH), 5.53–4.97 (m, 16H, –CH_2_–OH), 4.95–4.74 (m, 8H, –C–CH_2_–O), 4.61–4.34 (m, 8H, –CH_2_), 4.21–3.57 (m, 8H, –CH_2_), 3.40 (q, 16H, –CH_2_–NH–), 2.61 (qt, 16H, –COO–CH_2_–), 1.81 (s, 16H, –C–CH_2_–CH_2_–), 1.42 (d, 72H, –CH_3_), 1.32–0.93 (m, 89H, –CH_2_–, –Si–(CH_3_)_3_), 0.92–0.51 (m, 40H, –CH_2_–, –CH_3_), 0.07 (s, 6H, –Si–CH_3_).

#### Synthesis of discrete 2Azo-4F-2Azo-8Boc (triblock co-ligomer precursor)

In a 20 mL Schlenk tube, TBS-4F-N_3_ (500.00 mg, 0.26 mmol) and alkyne-2Azo-Br-4Boc (459.92 mg, 0.31 mmol) were dissolved in anhydrous THF, and then PMDETA (107.45 mg, 0.62 mmol) and CuBr (74.11 mg, 0.52 mmol) were added. The Schlenk tube was filled with argon three times to deoxygenate it and reacted for 48 h at room temperature. After the reaction, the reaction mixture was concentrated by rotary evaporation to remove THF, extracted with ethyl acetate and then purified by column chromatography. Then, TBAF was used to deprotect the TBS of TBS-4F-2Azo-Br-4Boc to obtain alkyne-4F-2Azo-Br-4Boc.

In a 20 mL Schlenk tube, alkyne-4F-2Azo-Br-4Boc (530.00 mg, 0.16 mmol) and TBS-2Azo-N_3_-4Boc (1070.00 mg, 0.69 mmol) were dissolved in anhydrous THF, and then PMDETA (168.00 mg, 0.97 mmol) and CuBr (57.4 mg, 0.40 mmol) were added. The Schlenk tube was filled with argon three times to deoxygenate it and reacted for 48 h at room temperature. After the reaction, the reaction mixture was concentrated by rotary evaporation to remove THF and then extracted with ethyl acetate. The organic phase was washed three times with brine, dried over Na_2_SO_4_ and concentrated *in vacuo*. The crude product was purified by column chromatography (silica gel, eluent: petroleum ether/THF = 1/1, v/v) to yield product 2Azo-4F-2Azo-8Boc as an orange-red solid (516.67 mg, 68.5%).^1^H NMR (300 MHz, CDCl_3_), *δ* (TMS, ppm): 8.42–8.15 (m, 7H, =CH–N–), 8.11–7.63 (m, 44H, ArH), 7.24–6.98 (m, 4H, ArH), 5.53–5.20 (m, 16H, –CH_2_–OH), 5.17 (d, 8H, –C–CH_2_–O), 4.99–4.73 (m, 8H, –CH_2_), 4.55 (d, 8H, –CH_2_), 3.43 (dd, 16H, –CH_2_–NH–), 2.62 (qd, 16H, –COO–CH_2_–), 1.73 (s, 16H, –C–CH_2_–CH_2_–), 1.51–1.33 (m, 72H, –CH_3_), 1.17 (d, 89H, –CH_2_–, –Si–(CH_3_)_3_), 0.89–0.52 (m, 40H, –CH_2_–, –CH_3_), 0.08 (d, 6H, –Si–CH_3_).

#### Synthesis of discrete 4(F-Azo)-8Boc (alternative co-oligomer precursor)

In a 25 mL Schlenk tube, TBS-Azo-N_3_-2Boc (969.00 mg, 1.14 mmol) and alkyne-F-NH_2_ (590.00 mg, 1.37 mmol) were dissolved in anhydrous THF, and then PMDETA (297.38 mg, 1.72 mmol) and CuBr (164.11 mg, 1.14 mmol) were added. The Schlenk tube was filled with argon three times to deoxygenate and reacted at room temperature. After reacting for 48 h, the reaction mixture was concentrated by rotary evaporation to remove THF, extracted with ethyl acetate and then purified by column chromatography to obtain TBS-Azo-2Boc-F-NH_2_.

Then, after using TBAF to remove the protective group TBS or using sodium azide to convert the amino group to azide, 4(F-Azo)-2Boc was synthesized by an iterative stepwise chain-growth approach of alkyne-Azo-2Boc-F-NH_2_ and TBS-Azo-2Boc-F-N_3_. In a 50 mL Schlenk tube, TBS-2(F-Azo)-N_3_-4Boc (304.10 mg, 0.12 mmol) and alkyne-2(F-Azo)-NH_2_-4Boc (383.10 mg, 0.16 mmol) were dissolved in anhydrous THF, and then PMDETA (41.60 mg, 0.24 mmol) and CuBr (17.20 mg, 0.12 mmol) were added. The Schlenk tube was filled with argon three times to deoxygenate it and reacted for 48 h at room temperature. After the reaction, the reaction mixture was concentrated by rotary evaporation to remove THF and then extracted with ethyl acetate. The organic phase was washed three times with brine, dried over Na_2_SO_4_ and concentrated *in vacuo*. The crude product was purified by column chromatography (silica gel, eluent: petroleum ether/tetrahydrofuran = 1/1, v/v) to yield an orange-red solid (358.50 mg, 61.4%). ^1^H NMR (300 MHz, CDCl_3_), *δ* (TMS, ppm): 8.22 (d, 7H, =CH–N–), 7.87 (dt, 39H, ArH), 7.00 (d, 9H, ArH), 5.56–5.17 (m, 16H, –CH_2_-OH), 5.01 (s, 9H, –C–CH_2_–O), 4.87 (d, 8H, –CH_2_), 4.55 (s, 8H, –CH_2_), 3.43 (s, 16H, –CH_2_–NH–), 2.62 (s, 16H, –COO–CH_2_–), 2.04 (dd, 16H, –C–CH_2_–CH_2_–), 1.54–1.36 (m, 72H, –CH_3_), 1.34–0.94 (m, 89H, –CH_2_–, –Si–(CH_3_)_3_), 0.96–0.51 (m, 40H, –CH_2_–, –CH_3_), 0.08 (d, 6H, –Si–CH_3_).

#### Synthesis of discrete co-oligomers 4F-4Azo-8NH_2_, 2Azo-4F-2Azo-8NH_2_ and 4(F-Azo)-8NH_2_ (removal of Boc protecting groups)

The Boc groups on the co-ligomer precursors of 4F-4Azo-8Boc, 2Azo-4F-2Azo-8Boc and 4(F-Azo)-8Boc were quantitatively removed by hydrolysis in the presence of trifluoroacetic acid (TFA) to obtain three sequence isomers of co-oligomers with amino group pendants of 4F-4Azo-8NH_2_, 2Azo-4F-2Azo-8NH_2_ and 4(F-Azo)-8NH_2_. Using 4F-4Azo-8NH_2_ as an example, the synthesis process was as follows: 4F-4Azo-8Boc (260.00 mg, 0.054 mmol) was dissolved in 1.0 mL of TFA, and after reacting at 25 °C for 12 h, the majority of TFA was removed by rotary evaporation. Then, the mixture was dropped into a saturated NaHCO_3_ aqueous solution, after which the liquid was removed *via* filtration. The sediment was washed with NaHCO_3_ aqueous solution and saturated NaCl solution until the pH became neutral. After centrifugal drying, a pale yellow solid of 4F-4Azo-8NH_2_ was obtained. The synthesis of 2Azo-4F-2Azo-8NH_2_ and 4(F-Azo)-8NH_2_ by hydrolysis in the presence of TFA is the same as that of 4F-4Azo-8NH_2_.

### Self-assembly of discrete co-oligomers in THF/water/HCl

The co-oligomer was first dissolved in THF solution and filtered through a 0.22 μm polytetrafluoroethylene filter. A clear transparent oligomer solution was obtained, and then quantitative Milli-Q water was added dropwise into the solution. Subsequently, hydrochloric acid aqueous solution was slowly dropped into the solution above. The assembled solution was sealed and aged by standing still at different times at room temperature.

### Preparation of samples for TEM testing

One drop of the self-assembled polymer solution was dropped onto the carbon support film-coated copper net and allowed to stand for 30 seconds, and then filter paper was used to draw the excess assembly solution along the edge. The assembly solution continued to drip and was repeated three times and left to dry under ambient conditions.

### Preparation of samples for AFM testing

The self-assembled polymer solution was dropped onto a precleaned silicon wafer and left to dry under ambient conditions.

### Preparation of samples for SEM testing

Sample preparation in the SEM experiment. The oligomer assembly solution of three different sequences was dropped onto precleaned tin foil paper and then put into a vacuum oven at 30 °C for quick drying.

### Preparation of samples for TEM testing after irradiation with UV light

The assembled solution was irradiated with UV light at 365 nm (with an intensity of 0.5 mW cm^−2^), and the TEM samples were prepared in a dark environment (refer to the preparation of TEM samples above) and wrapped in tin foil for storage away from light.

## Results and discussion

### Synthesis and characterization of discrete co-oligomers with different building blocks in the main chain

Two monomers, TBS-F-NH_2_ and TBS-Azo-Br-2Boc, were efficiently synthesized, containing fluorene and azobenzene structures, respectively, in the backbone in the synthetic strategy. Specifically, two amino groups protected by Boc are grafted at the side chain of the azobenzene monomer, and both monomers contain an identical alkynyl group that is protected by tert-butyldimethylsilyl (TBS), which can be removed in the presence of tetrabutylammonium fluoride (TBAF), and an amino group or a bromine atom that can be modified into an azide group. Subsequently, three co-oligomer precursors, 4F-4Azo-8Boc (diblock oligomer), 2Azo-4F-2Azo-8Boc (triblock oligomer) and 4(F-Azo)-8Boc (alternative oligomer), were efficiently synthesized by a stepwise growth strategy combined with protection-deprotection *via* an efficient CuAAC click reaction of azide–alkyne.^5^ Finally, the Boc groups were removed by hydrolysis in the presence of trifluoroacetic acid (TFA) to obtain sequence isomeric co-oligomers of 4F-4Azo-8NH_2_, 2Azo-4F-2Azo-8NH_2_ and 4(F-Azo)-8NH_2_ with amino group pendants. These molecular structures of oligomers are summarized in Scheme 1, and the synthetic routes and preparation process are described in the synthetic part above and ESI.†

**Fig. 1:**
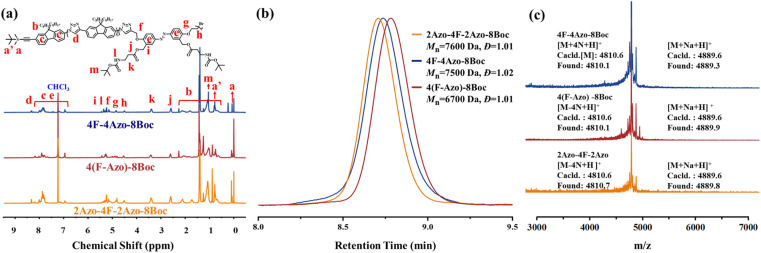
The characterization of co-oligomer precursors of 4F-4Azo-8Boc, 2Azo-4F-2Azo-8Boc and 4(F-Azo)-8Boc. (a) ^1^H NMR spectra, (b) GPC elution curves and (c) MALDI-TOF spectra for 2Azo-4F-2Azo-8Boc, 4(F-Azo)-8Boc and 4F-4Azo-8Boc respectively.

The three co-oligomer precursors 4F-4Azo-8Boc, 2Azo-4F-2Azo-8Boc and 4(F-Azo)-8Boc were characterized by ^1^H nuclear magnetic resonance (NMR), gel permeation chromatography (GPC) and matrix-assisted laser desorption ionization time-of-flight mass spectrometry (MALDI-TOF MS) (Fig. 1). As shown in the ^1^H NMR spectra (Fig. 1(a)), the typical protons of triazoles and aromatic rings (fluorene and azobenzene) and alkyl chains at the C9 position of fluorenes were clearly observed, and the corresponding integral values were in agreement with those obtained by calculation, confirming the co-oligomer structures. As shown in Fig. 1(b), the GPC elution curves presented a significantly low dispersity (*Đ* = 1.01–1.02). GPC measurement found molecular weights of oligomers higher than theoretical values because the rigid conjugation structures of the oligomers are stronger than that of styrene, which was used as the standard sample of GPC measurement. Notably, the three isomeric co-oligomers showed different elution times, suggesting that different semirigid conjugate backbones induced distinguished hydrodynamic volumes. The oligomer 4(F-Azo)-8Boc presented the longest elution time, showing that 4(F-Azo)-8Boc has the smallest hydrodynamic volume, which can be attributed to its relatively flexible structure compared with the other oligomers. In addition, it could be observed from the MALDI-TOF MS results that the *m*/*z* peaks of the three oligomers were almost identical, and the measured results matched well with the theoretical values (Fig. 1(c) and S5†). Notably, the molecular weights of the oligomers indicated loss of two, four, six, and eight nitrogen atoms, respectively, as shown in partially enlarged MALDI-TOF spectra (Fig. S5†), which can be attributed to an effect of the rigid fluorene unit that is directly connected to triazole rings resulting in the expulsion of N_2_ (two nitrogen atoms) from the triazole.^45^ The protective Boc groups could be completely removed in the presence of trifluoroacetic acid (TFA) to expose the amino groups.^31^^1^H NMR spectra of three co-oligomers before and after hydrolysis after hydrolysis were examined, that proton peaks of Boc groups of co-oligomers disappeared after hydrolysis compared with those before hydrolysis, indicating the complete removal of Boc groups (Fig. S6–S8†).

### Self-assembly behavior of discrete fluorene-azobenzene co-oligomers in solution

Amphiphilic block polymers with hydrophilic and hydrophobic chain segments maintain incompatibility in a mixture of common solvents and selective solvents. Driven by hydrophobic interactions and interchain attraction, hydrophobic chain segments undergo rearrangement along the direction of the molecular chain with the addition of selective solvents such as water and spontaneously form assemblies with specific structures.^16^ The self-assembly of fluorene-azobenzene co-oligomers was carried out in solution by adding hydrochloric acid Milli-Q water to the solution in THF very slowly. The amino groups of the azobenzene side chain were converted into a hydrophilic quaternary ammonium salt in the presence of hydrochloric acid, rendering the co-oligomers amphiphilic and causing spontaneous self-assembly in a mixed solution of THF/water/HCl.

The self-assembly behaviors of the co-oligomers in THF/water/HCl solution were investigated in a conventional approach as described below. With water and hydrochloric acid aqueous solutions added into the oligomer solution in THF in sequence, the oligomer assembled spontaneously into nanostructures, and the morphology and size of assembly aggregates were then measured by TEM, SEM, AFM and DLS. The external experimental factors were controlled, including the initial concentration of oligomer in THF (*C*_0_), the volume ratio of THF/water, the content of hydrochloric acid aqueous solution at a certain concentration, and the aging time that the assembled solution was sealed and left still at room temperature. Meanwhile, the water addition rate (0.2 mL h^−1^), assembly temperature (30 °C) and stirring speed (300 rpm) remained unchanged in the assembly process.

**Fig. 2:**
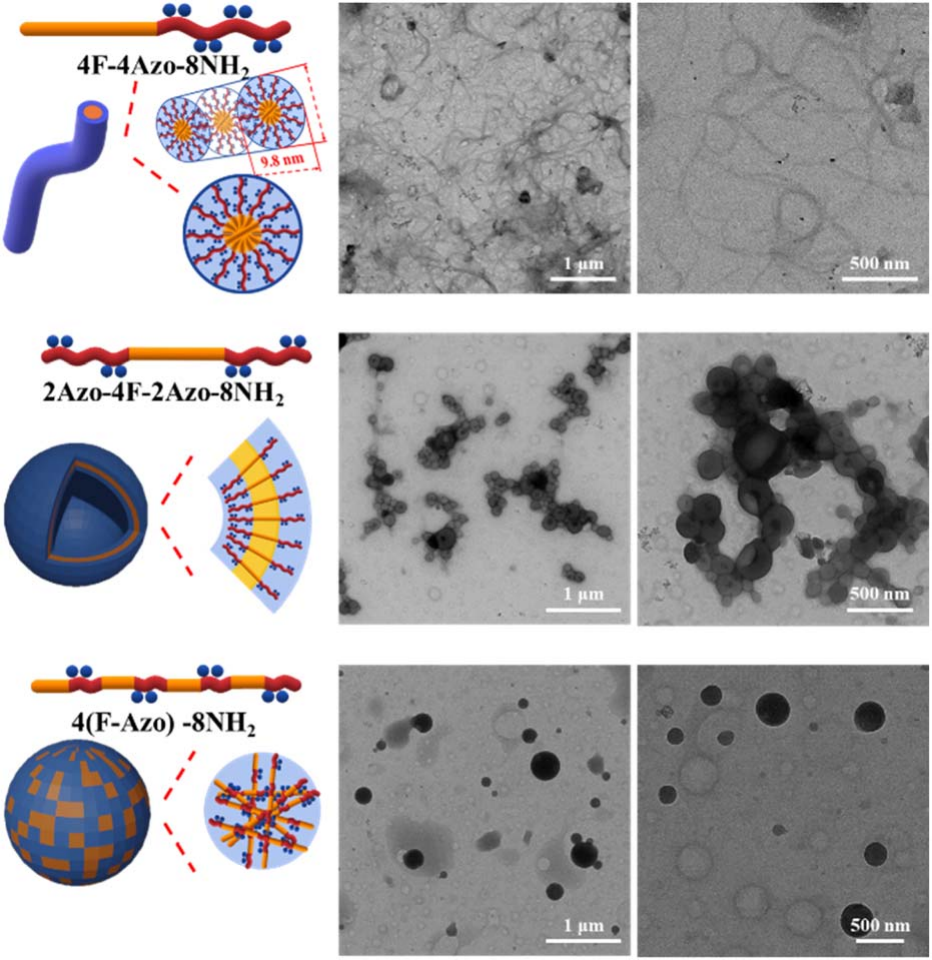
(Left) Schematic diagram of chemical structure of 4F-4Azo-8NH_2_, 2Azo-4F-2Azo-8NH_2_ and 4(F-Azo)-8NH_2_, and schematic illustration of molecular stacking and morphologies in self-assemblies in THF/water/HCl; (Right) morphologies of self-assemblies determined by TEM of 4F-4Azo-8NH_2_, 2Azo-4F-2Azo-8NH_2_ and 4(F-Azo)-8NH_2_ in THF/water/HCl. Assembly conditions: solvent ratio: THF/water/HCl (1.0 M) (v/v/v) = 1/0.5/0.4; initial concentration: *C*_0_ = 1.0 mg mL; injection speed: 0.2 mL h; temperature: 30 °C; stirring speed: 300 rpm; aging times for 1 h.

4F-4Azo-8Boc-8NH_2_ was first selected as a representative sample to precisely tailor the self-assembly conditions for fabricating well-defined nanoparticles. As shown in the TEM images in Fig. 2, 4F-4Azo-8Boc-8NH_2_ formed nanofibers after aging for 1 h under assembly conditions in which the concentration of hydrochloric acid aqueous solution was 1.0 M and volume ratio of THF/water/HCl (1.0 M) was 1/0.5/0.4 (v/v/v) at an initial concentration of 1.0 mg mL^−1^. As the water content gradually increased from 0.5 mL to 1.0 mL to 2.0 mL (Fig. S9(a)–(c)†), the nanofibers continued to aggregate, and hence, the regularity of the assembled morphology gradually declined. Eventually, large aggregates were formed at water contents of 1.0 mL to 2.0 mL (Fig. S9(b) and (c)†) due to the interaction of hydrophobic chain segments. It was noted that as the ratio of THF/water/HCl (v/v/v) remained identical (1/0.5/0.4); increasing the concentration appropriately of hydrochloric acid aqueous solution benefited the formation of thin long fibers within the concentration range from 0.5 M to 1.0 M (Fig. S9(a) and (d)†), while irregular aggregation was observed at higher concentrations (2.0 M or 4.0 M) (Fig. S9(e) and (f)†). Additionally, changes in the assembly morphology were observed as the aging time increased (Fig. S10(a)–(f)†). In the first 10 minutes of aging, the oligomers formed thin and short nanofibers (Fig. S10(a)†), and then these fibers became thread-like and tended to intertwine loosely with each other as the aging time extended to 1 h (Fig. 2(a) and S10(b)†), and this morphology remained virtually unchanged during the period up to 12 h (Fig. S10(d)†). As it was prolonged to 1 day, some nanofibers tightly wrapped each other to form large clumps (Fig. S10(e)†) and eventually presented large-scale clumps 3 days later (Fig. S10(f)†) due to the accumulation of winding fibers.

**Fig. 3:**
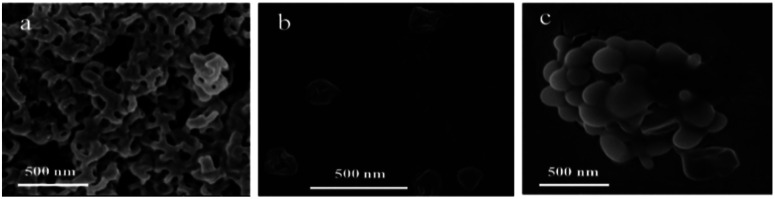
SEM images of self-assemblies of 4F-4Azo-8NH_2_ (a), 2Azo-4F-2Azo-8NH_2_ (b) and 4(F-Azo)-8NH_2_ (c). Assembly conditions: solvent ratio: THF/water/HCl (1.0 M) (v/v/v) = 1/0.5/0.4; initial concentration: *C*_0_ = 1.0 mg mL; injection speed: 0.2 mL h; temperature: 30 °C; stirring speed: 300 rpm; aging times for 1 h.

The assembly of the other co-oligomers 2Azo-4F-2Azo-8NH_2_ and 4(F-Azo)-8NH_2_ was implemented under the same experimental conditions as 4F-4Azo-8NH_2_ to contrast the self-assembly behavior of these sequence-defined isomers (*C*_0_ = 1.0 mg mL^−1^, THF/water/HCl (1.0 M) (v/v/v) = 1/0.5/0.4, speed of adding water: 0.2 mL h; temperature: 30 °C; stirring speed: 300 rpm, aging time for 1 h). Hollow vesicle structures could be clearly observed in the TEM images of 2Azo-4F-2Azo-8NH_2_, and the outer diameter of vesicles ranged from hundreds of nanometers to several micrometers (Fig. 2 and S12†). Meanwhile, many vesicle structures with a small size of approximately 10 nm were also noticed from partially enlarged TEM images (Fig. S11(b)†). The dynamic laser scattering (DLS) test results showed two peaks, of which the hydrodynamic diameters (*D*_h_) of one curve centred around 3–4 nm, while the *D*_h_ of the other was in a wide distribution between several hundred nanometers and several micrometers (Fig. S11(a)†). Based on the thickness of the vesicle wall obtained by both TEM and AFM analysis described below, these aggregates with very small sizes were not included in the vesicles, and they can be speculated to globular micelles formed by a single chain of the triblock co-oligomer. The DLS result was basically consistent with the TEM results, suggesting that larger vesicles might be formed by the fusion of smaller vesicles. The wall thickness of vesicles is an important parameter for studying the packing model of polymers in vesicles,^46^ and hence, it was measured by enlarged TEM images (Fig. S12†). The wall thicknesses of the wall appeared to change slightly in vesicles of different sizes, with average thicknesses of 4.4 nm to 4.5 nm. The alternating-structured isomer 4(F-Azo)-8NH_2_ formed a spherical micellar complex ranging from tens to hundreds of nanometers in diameter by the observation of TEM images (Fig. 2). Usually, alternating-structured amphipathic polymers with two coil alternating segments in the backbone adopt a folded-chain mechanism in the self-assemblies. Nevertheless, we suspect it to be difficult to fold for the alternating distribution of multiblock with rigid backbone, and hence the 4(F-Azo)-8NH_2_ assembled into spherical micellar complex with hydrophilic and hydrophobic chain segments arranged on the micelle surface.^47,48^ The self-assembled morphologies of the three amphipathic co-oligomer isomers above were also observed by scanning electron microscopy (SEM, Fig. 3) and atomic force microscopy (AFM, Fig. S13(a) and (b)†), showing loosely intertwining nanofibers, hollow vesicles and large compound micelles, which were consistent with the TEM results.

Although these oligomers possess identical compositions, the sequence isomerism at the ionic side chain of the monomer leads to distinguished self-assembly behaviors. To gain insight into the packing conformations and mechanism in self-assembly, the co-oligomers were investigated by differential scanning calorimetry (DSC), X-ray scattering (XRD), and UV/vis absorption spectroscopy. All oligomers showed obvious melting peaks at approximately 95–100 °C in the second DSC heating curves, whereas every isomer presented different double peaks, indicating their different internal stacking structures (Fig. S14†). The XRD of co-oligomers was investigated by using fluorene block 4F as a contrast. Compared with the XRD spectra (Fig. S15†), fluorene block 4F exhibited a very weak diffraction peak, while the co-oligomers of imbedded azobenzene building blocks presented distinctly distinguished diffraction peaks, suggesting that the co-oligomers possess crystallinity behavior.^49^ In contrast, the XRD patterns of 4F-4Azo-8Boc-8NH_2_ presented distinctly sharp peaks at 2*θ* = 20° and 2*θ* = 40° compared with the other two, showing a more ordered stacking structure for the diblock co-oligomer than for the triblock and multiple block isomers. This may promote the formation of relatively ordered 1D nanostructures such as nanofibers in the process of self-assembly of 4F-4Azo-8NH_2,_ as presented above.

**Fig. 4:**
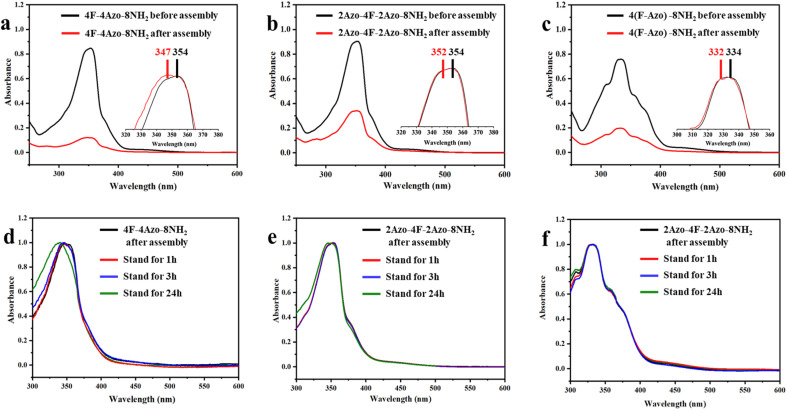
UV-vis absorption spectra of 4F-4Azo-8Boc-8NH_2_ (a), 2Azo-4F-2Azo-8NH_2_ (b) and 4(F-Azo)-8NH_2_ (c) in THF and in THF/water/HCl after self-assembly (aging times for 1 h); the insets in (a)–(c) are normalized UV-vis absorption spectra; normalized UV-vis absorption spectra of 4F-4Azo-8Boc-8NH_2_ (d), 2Azo-4F-2Azo-8NH_2_ (e) and 4(F-Azo) -8NH_2_ (f) of assembled solution above at aging different times. Assembly conditions: solvent ratio: THF/water/HCl (1 M) (v/v/v) = 1/0.5/0.4; initial concentration: *C*_0_ = 1.0 mg mL; adding water speed: 0.2 mL h; temperature: 30 °C; stirring speed: 300 rpm.

The co-oligomers before and after assembly were investigated by UV/vis spectroscopy to ascertain intermolecular stacking. The absorption intensities at approximately 330 nm corresponding to the π–π* transition of *trans*-azobenzene in all assembly solutions of co-oligomers (aging times for 1 h) decreased obviously compared with those of oligomers in THF before assembly, indicative of intermolecular π–π stacking of azobenzene moieties in assembled aggregates. Furthermore, the maximal absorption peaks in the assembled solution exhibited a small blueshift, with 4F-4Azo-8NH_2_ exhibiting a relatively large shift, from 347 nm to 354 nm (Fig. 4(a)–(c)). With increasing aging times of the assembled solution from 1 h to 24 h, the absorption peaks showed a further blueshift, of which 4F-4Azo-8Boc-8NH_2_ had an obvious change in the maximal absorption wavelength (Fig. 4(d)–(f)). This indicates that the oligomer molecules prefer to assemble *via* H-aggregation, as the parallel orientation of the azobenzene moieties in such an arrangement mode could produce higher orderliness of stacking in some directions.^50,51^ This is consistent with the differences in stacking structures among the three co-oligomers in the above analysis, further showing that the diblock co-oligomer has a more ordered stacking structure.

As discussed above, TEM images of the 4F-4Azo-8NH_2_ self-assembly showed that some tiny and short fibers were distributed near the long and crude fibers. We speculate that the long and crude nanofibers evolved from the winding and accumulation of tiny and short fibers. The lengths of the main chain in the most stable state of 4F-4Azo-8NH_2_ were calculated by theoretical simulation to be 11.7 nm, of which the azobenzene block with amino side-chain groups is approximately 6.8 nm, and the fluorene block is 4.9 nm, as shown in the detailed image (Fig. S16†). We hypothesize that the diblock oligomer 4F-4Azo-8Boc-8NH_2_ tended to be close to each other and organized by face-to-face stacking, leading to the formation of fibers with fluorene blocks as the core and strong hydrophilic quaternary ammonium salt as the shell. As observed by AFM (Fig. S13(a) and (c)†), the height of these tiny fibers was approximately 9.8 nm, which is less than the theoretical value ((6.8 × 2 + 4.9) = 18.5 nm) obtained by calculation, possibly due to collapse of assembly aggregate under the conditions of the AFM experiment. In the case of triblock oligomer 2Azo-4F-2Azo-8NH_2_, the height of collapsed vesicles was tested to be 9.8 nm (Fig. S13(b) and (d)†); that is, the thickness of the vesicle wall was 4.9 nm, which is substantially close to the value in TEM testing (4.5 nm) as described above and is also consistent with the theoretical length of the fluorene block (4.9 nm) (Fig. S17†). Thus, we speculate that the triblock co-oligomer stacked together into monolayers and hence formed vesicles instead of fibers, having a sandwich structure with a hydrophobic fluorene block as the core and two layers of hydrophilic azobenzene blocks as the shell. For the alternative-type oligomer 4(F-Azo)-8NH_2_, a special alternating structure tended to agglomerate, in which a strong hydrophilic quaternary ammonium salt can provide better solubility; consequently, it formed a micellar complex. According to the experimental and theoretical results, we can propose reasonable molecular stacking and morphological evolution of aggregates of three block co-oligomer isomers during self-assembly in a mixed solution of THF/water/HCl, as displayed in Fig. 2 and S16–S18.†

### Photoresponsive behavior of assembly aggregates of co-oligomers

**Fig. 5:**
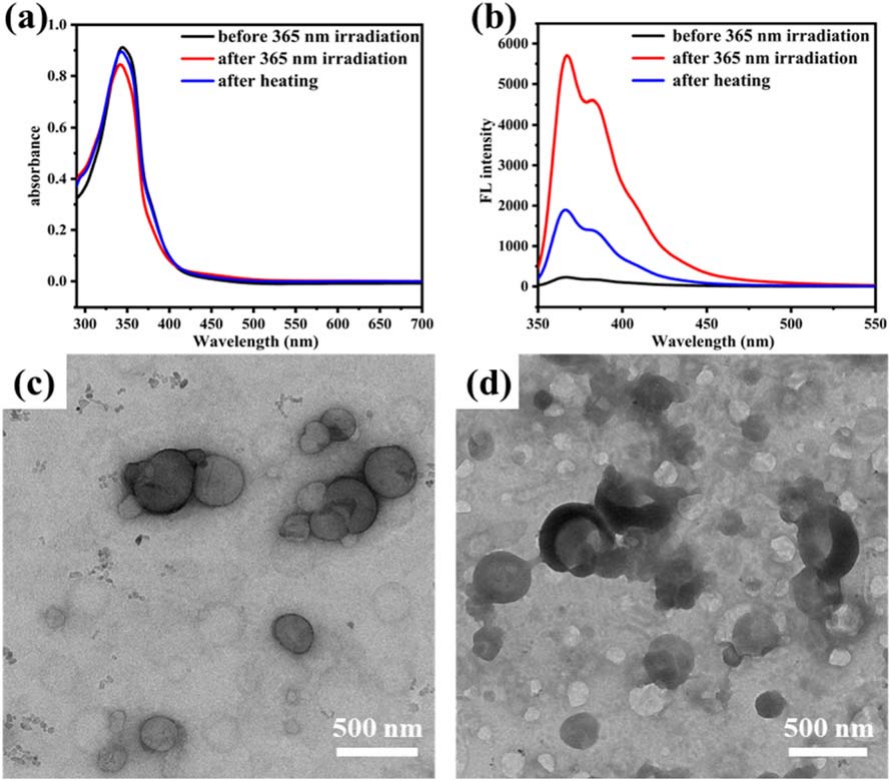
UV-vis absorption spectra, fluorescence emission spectra and TEM images of assembled solutions of 2Azo-4F-2Azo-8NH_2_ in THF/water/HCl (1.0 M) before and after light irradiation with 365 nm (0.5 mW cm^−2^), and after heating recovery at about 60 °C for an hour. (a) UV-vis absorption spectra before and after light irradiation with 365 nm, and after heating recovery; (b) fluorescence emission spectra before and after light irradiation with 365 nm, and after heating recovery, excitation wavelength: 320 nm; (c) TEM images of self-assembled aggregates in the initial state; (d) TEM images of self-assembled aggregates after 365 nm light irradiation.

It is well known that azobenzene can undergo reversible *cis*–*trans* isomerization under light irradiation or heating, accompanied by changes in the shape and polarity of the molecule. Here, 2Azo-4F-2Azo-8NH_2_ was selected as a representative sample to further investigate the photoresponsive features of the oligomer nanoparticle by UV light irradiation at 365 nm and heating recovery. The UV-vis absorption of the assembled solution of 2Azo-4F-2Azo-8NH_2_ in the initial state and after light irradiation and heating recovery were recorded by UV-vis spectrometry, as shown in Fig. 5(a). The absorption peak at approximately 330 nm corresponding to the π–π* transition of *trans*-azobenzene decreased obviously after UV light irradiation, implying that azobenzene in oligomer aggregates had undergone *trans*-to-*cis* photoisomerization. Subsequently, the sample after UV light irradiation was heated at about 60 °C for an hour for the recovery of *cis*-to-*trans* isomerization of azobenzene. It was noted that the absorption intensity at approximately 330 nm returned to the original state after heating, showing azobenzene reverting to *trans*-ones from *cis*-isomers. The morphological transformation of the aggregates under light irradiation was ascertained by TEM. As expected, it was observed from TEM images (Fig. 5(c) and (d)) that the vesicle structures were destroyed and became irregular aggregates after UV light irradiation, which can be attributed to planar *trans*-azobenzene transforming into nonplanar *cis*-azobenzene under UV light irradiation, leading to a change in the conformation and polarity of the oligomers. Furthermore, no fluorescence of the assembled solutions was observed before light irradiation, whereas obvious fluorescent emission was observed after light irradiation due to *trans*-to-*cis* photoisomerization. Meanwhile, the intensity of fluorescence was greatly reduced after heating due to azobenzene reverting to *trans*-ones from *cis*-isomers (Fig. 5(b)). Plane *trans*-azobenzene usually shows a quenching effect on the fluorescence of fluorophores, mainly due to photoinduced electron transfer (PET) or twisted intermolecular charge transfer (TICT).^52–54^ However, nonplanar *cis* azobenzene can restrain π electronic transmission in the N=N bond of azobenzene, leading to fluorescence enhancement after azobenzene undergoes *trans*-to-*cis* isomerization.^55^

## Conclusions

In summary, first, two types of monomers comprising azobenzene units and fluorene units in the backbone were reasonably designed and synthesized. Then, sequence-controlled three block co-oligomer isomers with a fluorene-azo block semirigid backbone, diblock type, triblock type and alternative type named 4F-4Azo-8NH_2_, 2Azo-4F-2Azo-8NH_2_ and 4(F-Azo)-8NH_2_ were achieved *via* iterative stepwise growth combined with a protection-deprotection strategy utilizing an efficient copper(i)-catalyzed alkyne–azide cycloaddition (CuAAC) reaction. These co-oligomers, which possess two primary amine groups on the side chain of each azobenzene unit, became hydrophilic quaternary ammonium salts when protonated in acidic aqueous solution and hence can be assembled in THF/water/HCl mixed solution. The sequence-controlled three block co-oligomer isomers assembled into nanofibers, hollow vesicles and spherical micellar complexes under the same assembly conditions. This demonstrates that sequence control can have a dramatic impact on the self-assembly of the co-oligomer. UV-vis absorption spectra, DSC and X-ray experiments combined with theoretical calculations reveal that contrasting differences in assembly morphology between three block co-oligomer isomers are due primarily to different molecular packing conformations and arrangement modes of conjugated building blocks of azobenzene and fluorene in the aggregates. Furthermore, the oligomer nanoparticles in the assembled solution demonstrate photoresponsive morphological transformation and fluorescence emission under UV light irradiation and heating recovery due to *trans*–*cis* photoisomerization of azobenzene. We expect this work to deliver guidance for customizing multilayer functional nanoparticles with various applications in supramolecular self-assembly.

## Author contributions

L. Ye and M. Liu contributed equally to this work.

## Conflicts of interest

There are no conflicts to declare.

## Supplementary Material

RA-013-D3RA04205G-s001
