# Supplementary material for: Sequence effect on the self-assembly of discrete amphiphilic co-oligomers with fluorene-azobenzene semirigid backbones
Source: RSC Adv. 2023 Aug 11;13(35):24181–90. doi: 10.1039/d3ra04205g (PMC10416705; doi:10.1039/d3ra04205g)
Supplement: RA-013-D3RA04205G-s001 [file RA-013-D3RA04205G-s001.pdf]

Electronic Supplementary Material (ESI) for RSC Advances.
This journal is © The Royal Society of Chemistry 2023

Supporting Information

Sequence effect on self-assembly of discrete amphiphilic copolyoligomers with fluorene-azobenzene semirigid backbones

Liandong Ye, § Min Liu, § Xiao Wang, Zhihong Yu, Zhihao Huang, Wei Zhang, Nianchen Zhou,* Zhengbiao Zhang* and Xiulin Zhu

Suzhou Key Laboratory of Macromolecular Design and Orecision Synthesis, Jiangsu Key Laboratory of Advanced Functional Polymer Design and Application, State and Local Joint Engineering Laboratory for Novel Functional Polymeric Materials, College of Chemistry, Chemical Engineering and Materials Science, Soochow University, Suzhou, 215123, P. R. China

[§]Authors L. Ye and M. Liu contributed equally to this work.

Supporting Information for this article is given via a link at the end of the document.

E-mail: nczhou@suda.edu.cn; zhangzhengbiao@suda.edu.cn

Experimental Procedures

Synthesis

Synthesis of the fluorene monomer TBS-F-NH_2 and azobenzene monomer TBS-Azo-Br-2Boc.

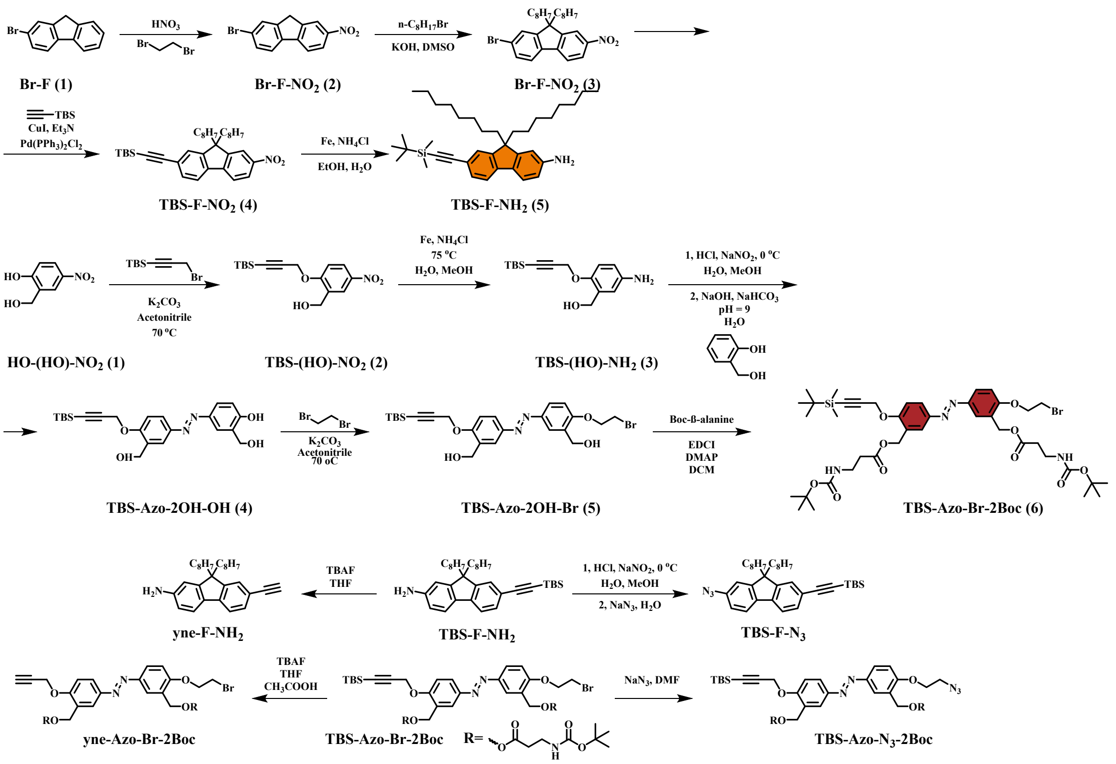

Scheme S1. The synthetic routes of fluorene monomer TBS-F-N_3 and azobenzene monomer TBS-Azo-Br-2Boc.

The synthetic routes of the both monomers of fluorene TBS-F-NH_2 and azobenzene TBS-Azo-Br-2Boc were shown in **Scheme S1**, and synthetic process was conducted according to a previous literature.[S1, S2] TBS-F-N_3 was obtained as a brown solid. ^1H NMR (300 MHz, $CDCl_3$) δ (TMS, ppm): 7.60-7.10 (m, 4H, ArH), 6.91-6.66 (m, 2H, ArH), 1.8-1.61 (m, 4H, -C-CH_2-CH_2-), 1.09-0.73 (m, 29H, -CH2-, -Si-$(CH_3)_3$), 0.68-0.54 (m, 6H, -CH_3), 0.34 (d, 4H, -CH_2-), 0.07-0.05 (m, 6H, -Si-CH_3). ^1H NMR spectrum and LCMS of TBS-F-N_3 are presented in Figure S1.

TBS-Azo-Br-2Boc was producted to yield as a yellow solid.^1H NMR (300 MHz, $CDCl_3$), δ (TMS, ppm): 7.90-7.70 (m, 4H, ArH), 7.07 (d, 1H, ArH), 6.87 (d, 1H, ArH), 5.17 (d, 4H, -CH_2-OH), 5.06 (s, 2H, -C-CH_2-O), 4.73 (s, 2H, -CH_2), 4.30 (t, 2H, -CH_2), 3.33 (q, 4H, -CH_2-NH), 2.52 (td, 4H, -COO-CH_2-), 1.32 (s, 18H, -CH_3), 0.79 (s, 9H, -Si-$(CH_3)_3$), 0.08 (s, 6H, -

Si-CH3). ^{1}H NMR spectrum is presented in Figure S2.

Synthesis of fluorene blocks (TBS-2F-NH_2, TBS-4F-NH_2) and azobenzene blocks (TBS-2Azo-Br-4Boc, TBS-4Azo-Br-8Boc)

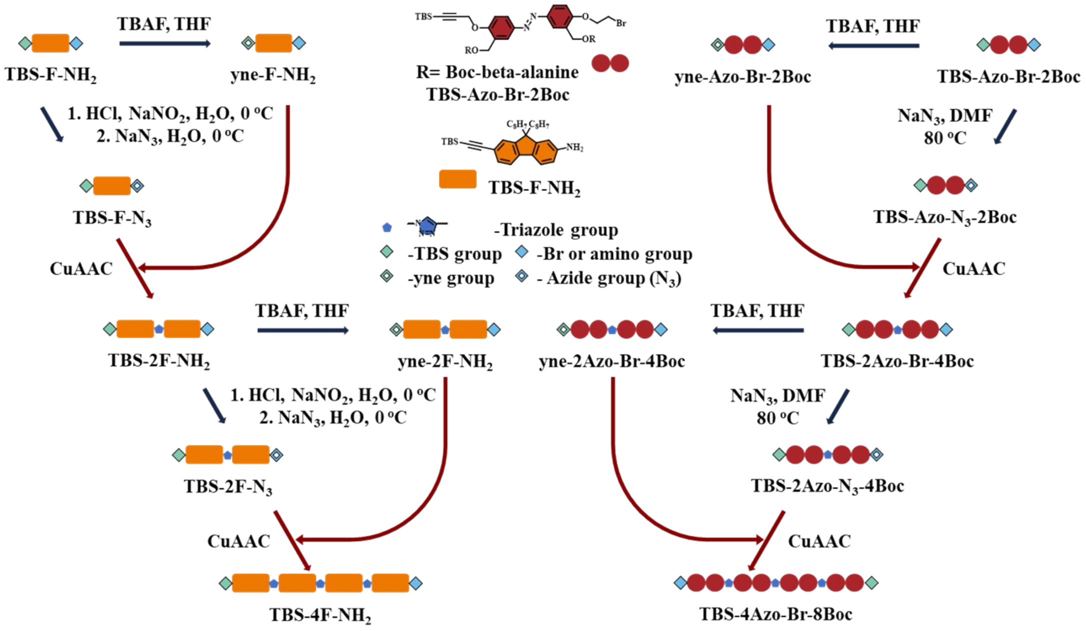

Scheme S2. Schematic diagram of the synthetic routes of fluorene blocks, TBS-2F-NH_2 and TBS-4F-NH_2, and azobenzene blocks, TBS-2Azo-Br-4Boc and TBS-4Azo-Br-8Boc.

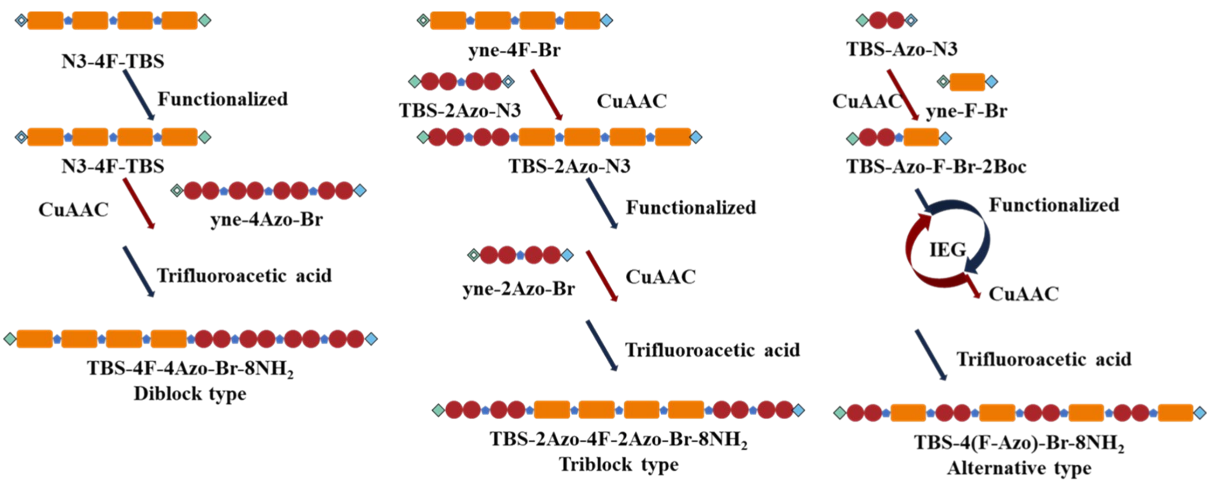

Scheme 3. Schematic diagram of synthetic routes of three sequence isomeric oligomers of 4F-4Azo-8NH_2, 2Azo-4F-2Azo-8NH_2 and 4(F-Azo)-8NH_2

Synthesis of TBS-2F-NH_2 and TBS-4F-NH_2

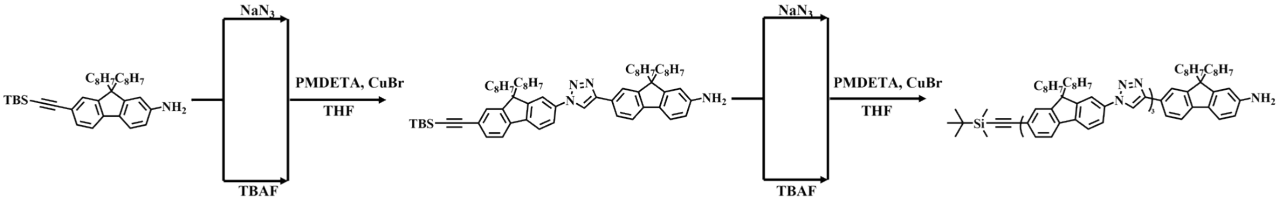

Scheme S4. The synthetic routes of fluorene blocks TBS-2F-NH_2 and TBS-4F-NH_2

Synthesis of azobenzene blocks TBS-2Azo-Br-4Boc and TBS-4Azo-Br-8Boc

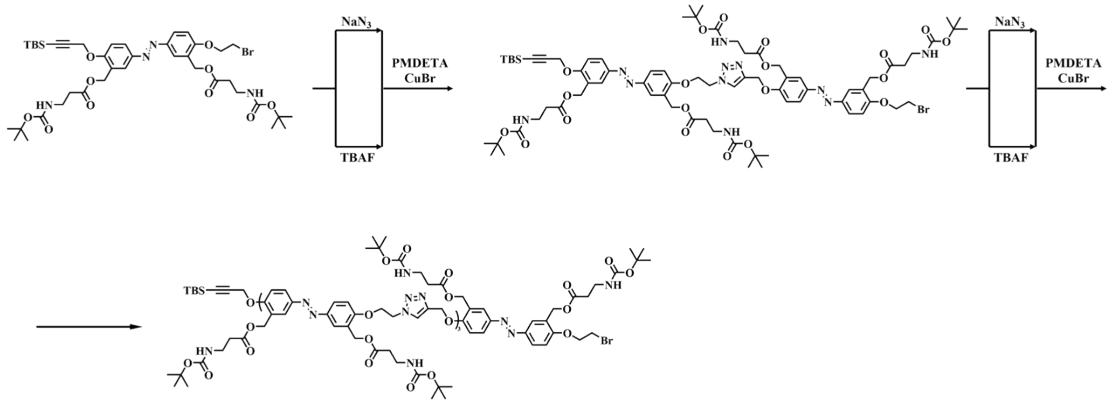

Scheme S5. Synthetic route of TBS-2Azo-Br-4Boc and TBS-4Azo-Br-8Boc.

Synthesis of TBS-2Azo-Br-4Boc and TBS-4Azo-Br-8Boc

^{1}H NMR spectra, MALDI-TOF spectra of TBS-2Azo-Br-4Boc and TBS-4Azo-Br-8Boc are presented in Figures S3(a) and (b), and GPC elution curves of TBS-Azo-Br-2Boc, TBS-2Azo-Br-4Boc and TBS-4Azo-Br-8Boc are presented in Figures S3(c), and IR spectra of TBS-Azo-Br-4Boc, TBS-2Azo-Br-4Boc and TBS-4Azo-Br-4Boc before and after azidation are presented in Figures S3(d).

Additional Results

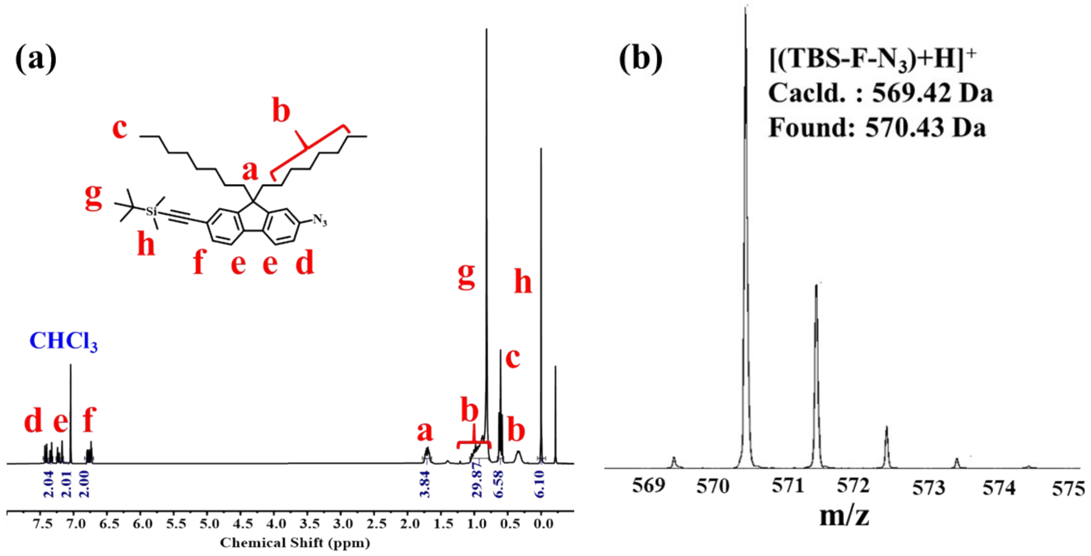

Figure S1. (a) ^{1}H NMR spectrum of TBS-F-N_3 in $CDCl_3$, (b) LCMS of TBS-F-N_3.

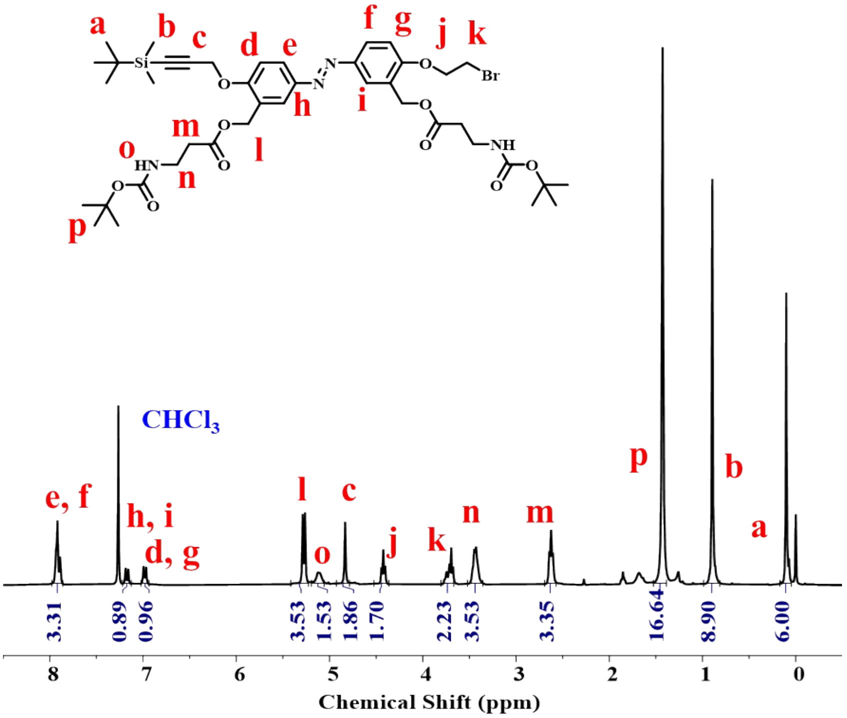

Figure S2. ^{1}H NMR spectrum of TBS-Azo-Br-2Boc in $CDCl_3$.

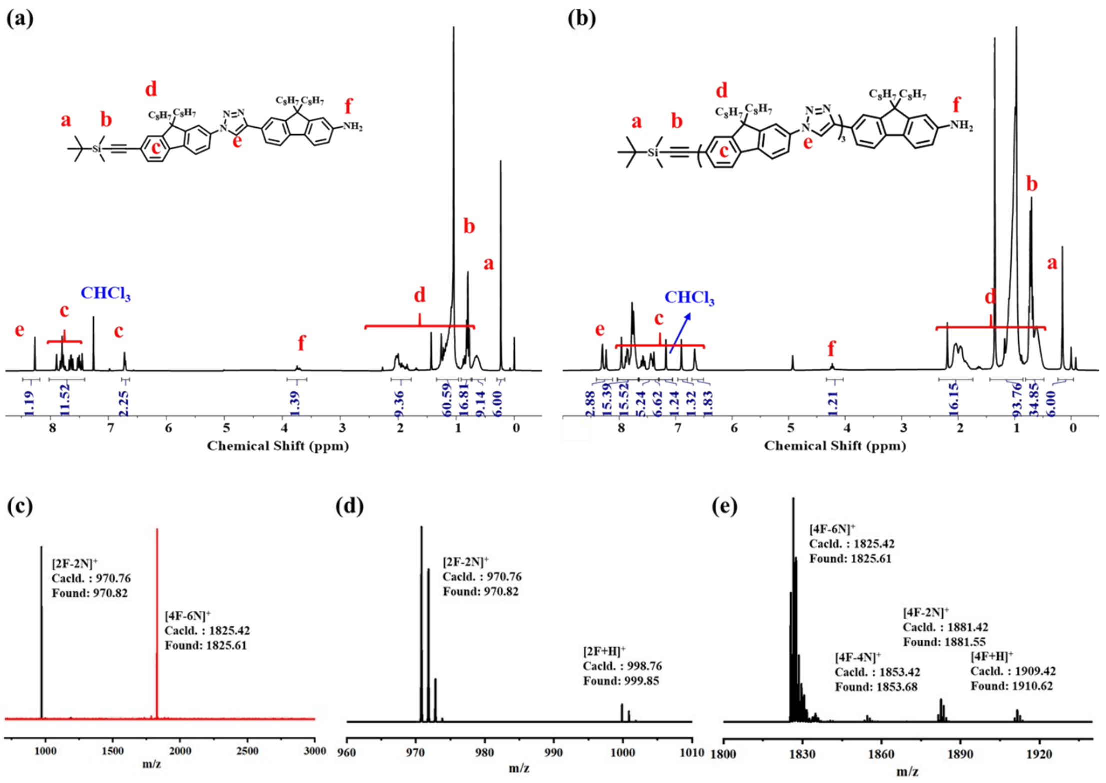

Figure S3. (a) ^{1}H NMR spectra of TBS-2F-NH_2 and TBS-4F-NH_2; (b) MALDI-TOF spectra of TBS-2F-NH_2 and TBS-4F-NH_2; (c) and (d) locally enlarged MALDI-TOF spectra of TBS-2F-NH_2 and TBS-4F-NH_2

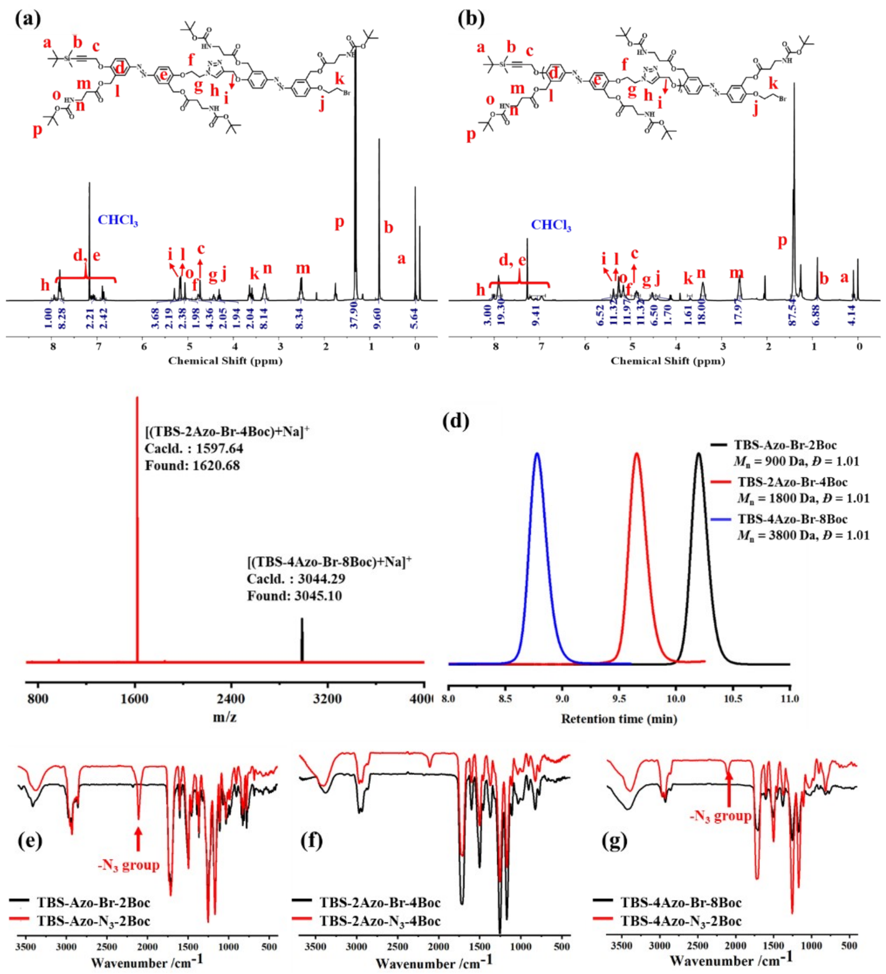

Figure S4. (a) ^{1}H NMR spectra of TBS-2Azo-Br-4Boc and TBS-4Azo-Br-8Boc in $CDCl_3$; (b) MALDI-TOF spectra of TBS-2Azo-Br-4Boc and TBS-4Azo-Br-8Boc; (c) GPC elution curves of TBS-Azo-Br-2Boc, TBS-2Azo-Br-4Boc and TBS-4Azo-Br-8Boc.(d) IR spectra of TBS-Azo-Br-2Boc, TBS-2Azo-Br-4Boc, TBS-4Azo-Br-8Boc before and after azidation.

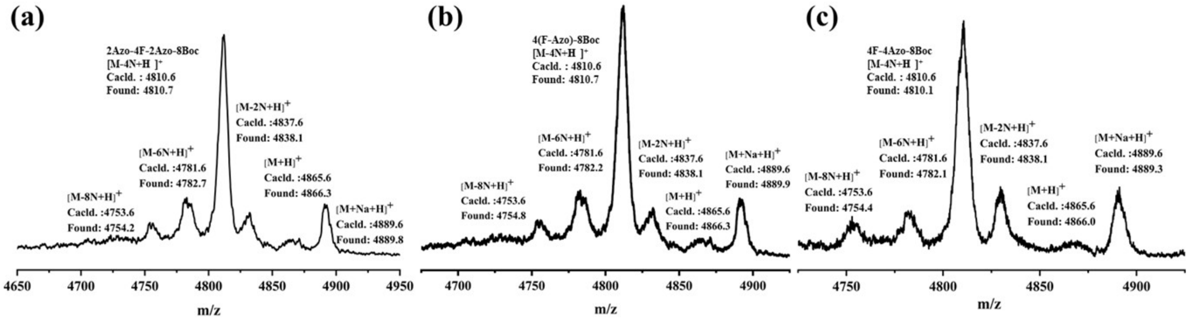

Figure S5. Partially enlarged MALDI-TOF spectra of (a) 2Azo-4F-2Azo-8Boc, (b) 4(F-Azo)-8Boc, (c) 4F-4Azo-8Boc.

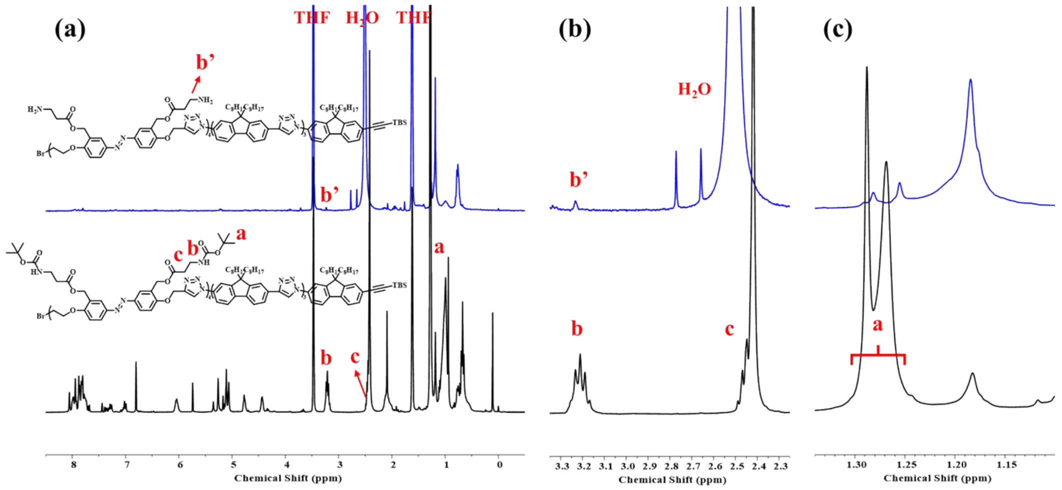

Figure S6. (a) ^{1}H NMR spectra of 4F-4Azo-8Boc and 4F-4Aazo-8NH$_2$ before and after hydrolysis in THF-d_8 and enlarged spectra in the range of (b) 3.3 ppm -2.3 ppm and (c)1.4 ppm -1.1 ppm.

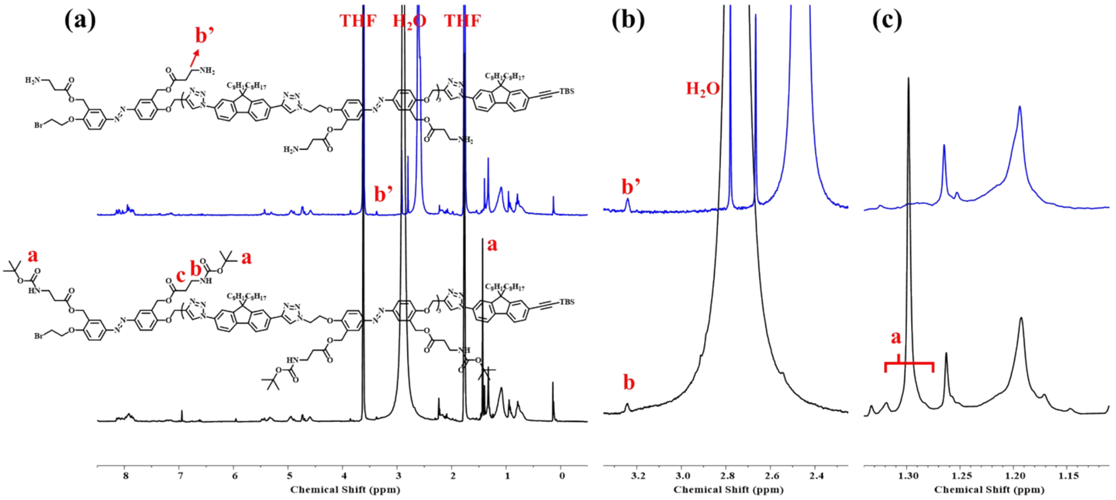

Figure S7. (a) ^{1}H NMR spectra of 4(F-A)-8Boc and 4(F-A)-8NH_2 before and after hydrolysis in THF-d_8 and enlarged spectra in the range of (b) 3.3 ppm -2.3 ppm and (c)1.4 ppm -1.1 ppm.

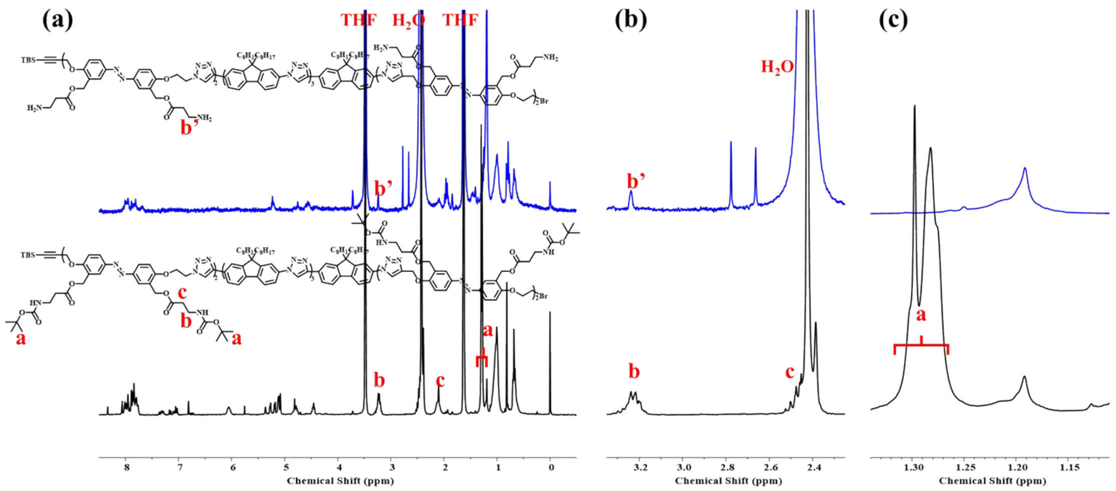

Figure S8. (a) ^{1}H NMR spectra of 2Azo-4F-2Azo-8Boc and 2Azo-4F-2Azo-8NH_2 before and after hydrolysis in THF-d_8 and enlarged spectra in the range of (b) 3.3 ppm -2.3 ppm and (c)1.4 ppm -1.1 ppm.

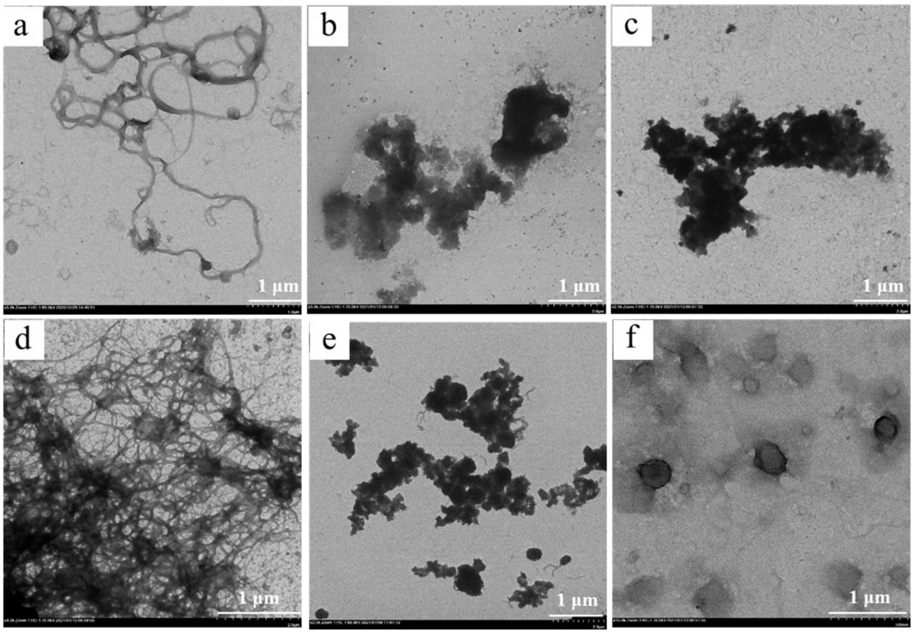

Figure S9. TEM images of assemblies of 4F-4Azo-8NH_2 assembled in THF/water/HCl systems with different volume fraction of THF/water/HCl and the concentration of hydrochloric acid aqueous solution. Assembly conditions: solvent ratio: (a) THF/water/HCl (1.0 M) (v/v/v) = 1:0.5:0.4, (b) THF/water/HCl (1.0 M) (v/v/v) = 1: 1: 0.4, (c) THF/water/HCl(1.0 M) (v/v/v) = 1: 2: 0.4; (d) THF/water/HCl (0.5 M) (v/v/v) = 1: 0.5: 0.4, (e) THF/water/HCl (2.0 M) (v/v/v) = 1:0.5:0.4, (f) THF/water/HCl (4.0 M) (v/v/v) = 1:0.5:0.4. Initial concentration: C_0 = 1.0 mg/mL; drop speed: 0.2 mL/h; temperature: 30 ^oC; stirring speed: 300 r/min, standing still for 1 h.

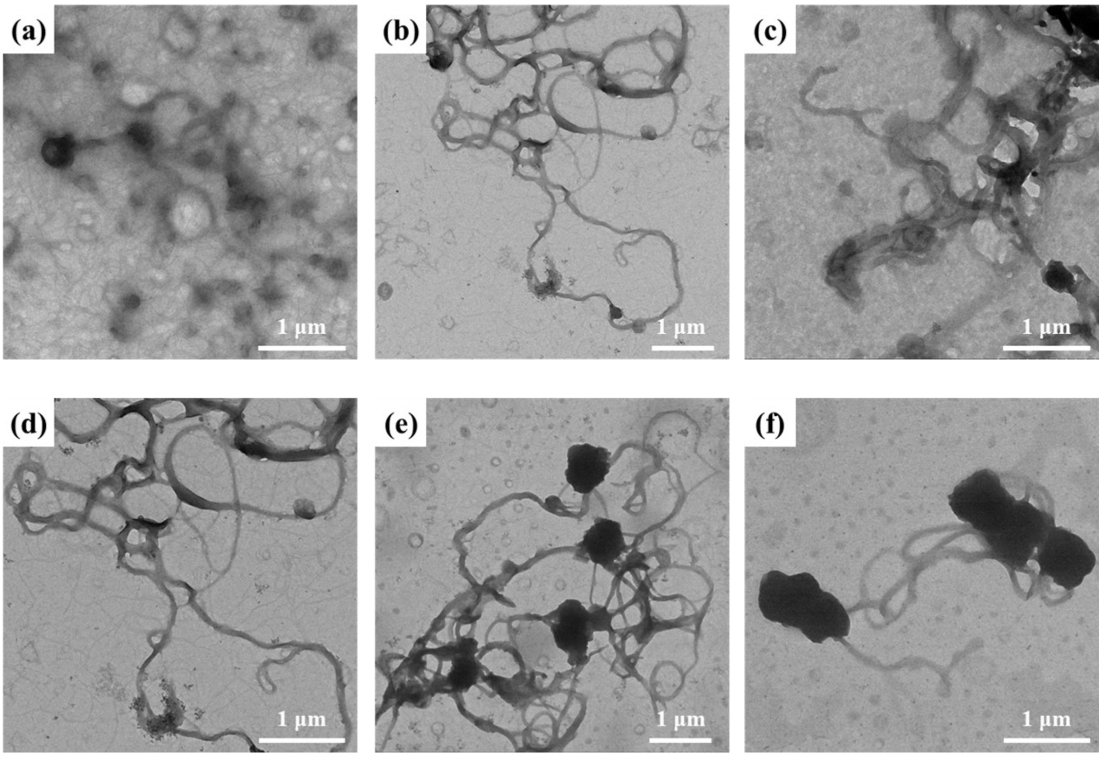

Figure S10. TEM images of the assemblies of 4F-4Azo-8NH_2 in THF/water/HCl (1.0M) at different aging times after completion of assembly. (a) 10 min, (b) 1 h, (c) 2 h, (d) 12 h, (e) 1day, (f) 3 days. Assembly conditions: solvent ratio: THF/water/HCl (1.0M) (v/v/v) = 1/0.5/0.4; initial concentration: C_0 = 1.0 mg/mL; injection speed: 0.2 mL/h; temperature: 30 ^{o}C; stirring speed: 300 r/min.

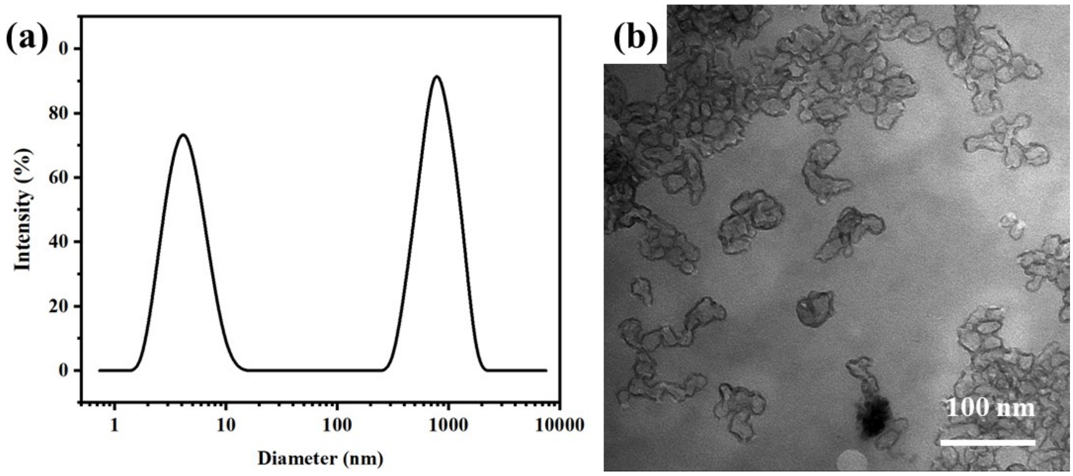

Figure S11. DLS curves of the assemblies of 2Azo-4F-2Azo-8NH_2 (a) and partial enlarged TEM images of smaller vesicles (b). Assembly conditions: solvent ratio: THF/water/HCl $_{(1M)}$ (v/v/v) = 1/0.5/0.4; initial concentration: C_0=1.0 mg/mL; drop speed: 0.2 mL/h; temperature: 30 ^{o}C; stirring speed: 300 r/min.

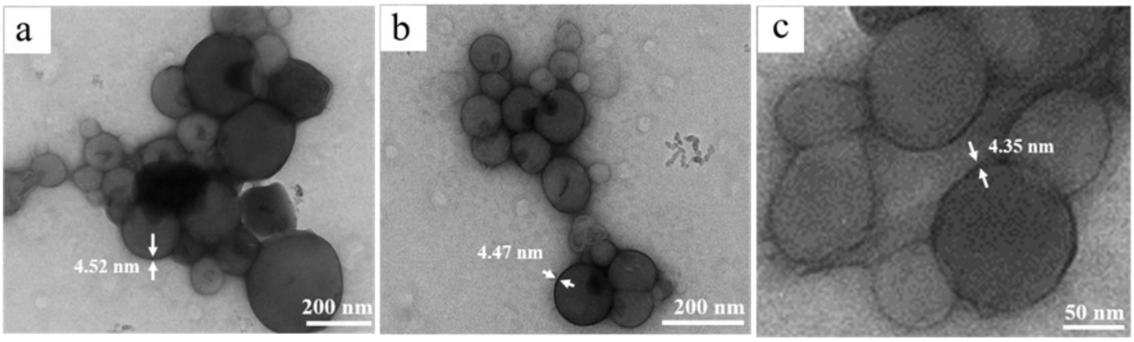

Figure S12. Enlarged TEM image of typical vesicles formed by 2Azo-4F-2Azo-8NH_2. Assembly conditions: solvent ratio: THF/water/HCl (1.0 M) (v/v/v) = 1/0.5/0.4; initial concentration: C_0=1.0 mg/mL; drop speed: 0.2 mL/h; temperature: 30 ^{o}C; stirring speed: 300 r/min.

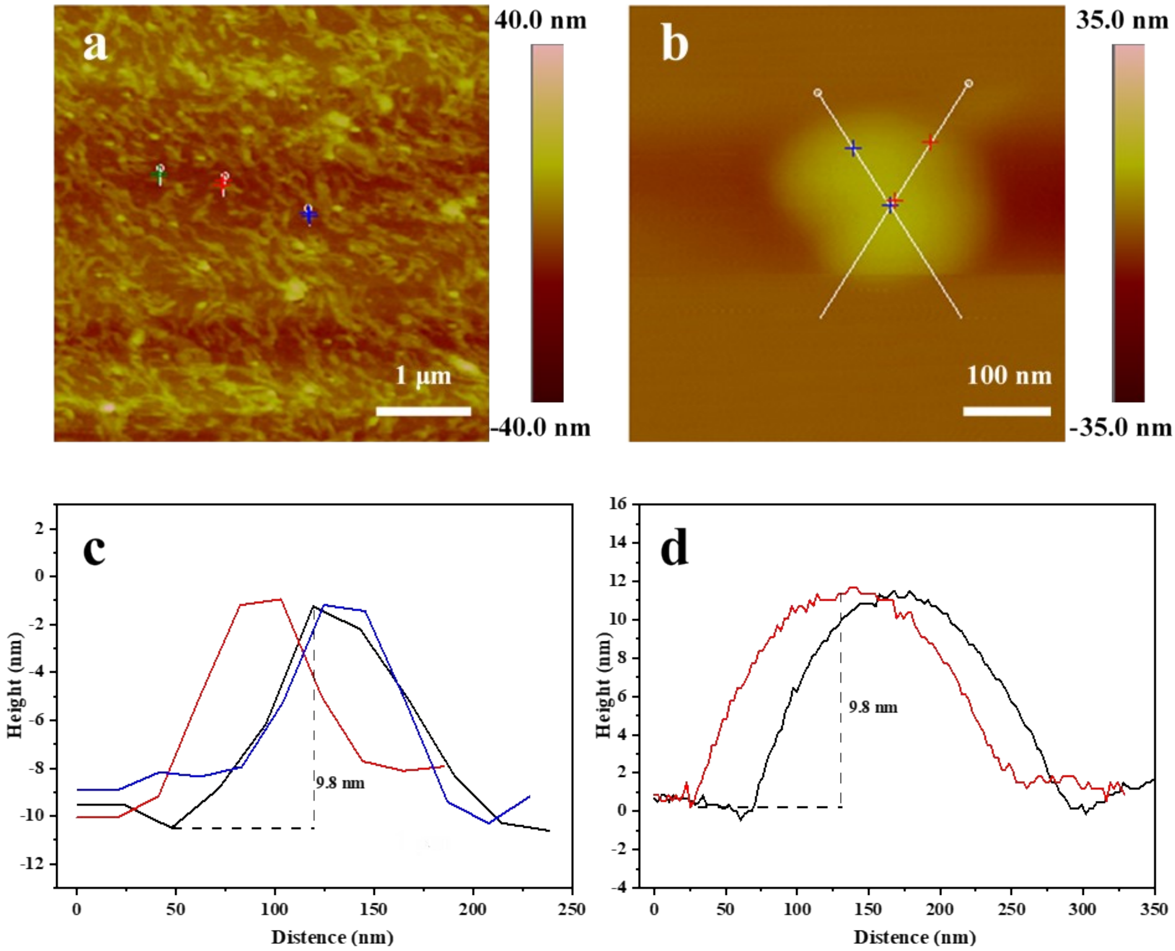

Figure S13. AFM images and height profile of the tiny fibers and vesicles obtained separately by assembly of 4F-4Azo-8NH_2 (a, c) and 2Azo-4F-2Azo-8NH_2 (b, d). Solvent ratio: THF/water/HCl (1.0 M) (v/v/v) = 1/0.5/0.4; initial concentration: C_0 = 1.0 mg/mL; drop speed: 0.2 mL/h; temperature: 30 ^{o}C; stirring speed: 300 r/min.

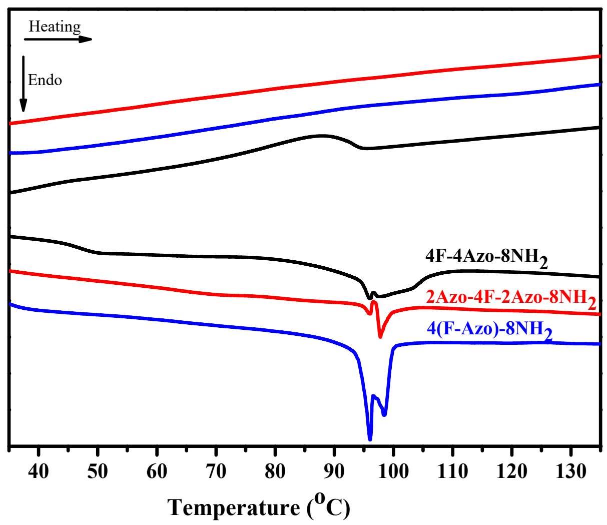

Figure S14. Second DSC heating curves of 4F-4Azo-8NH$_2$, 2Azo-4F-2Azo-8NH$_2$ and 4(F-Azo)-8NH$_2$ with a heating rate of 10 ^{o}C/min in N$_2$.

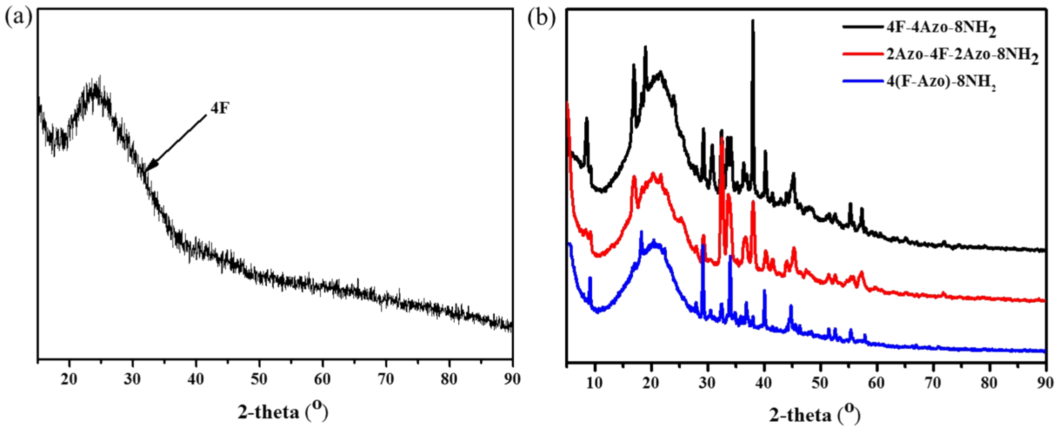

Figure S15. XRD spectra of 4F (a) and oligomers 4F-4Azo-8NH$_2$, 2Azo-4F-2Azo-8NH$_2$, 4(F-Azo)-8NH$_2$ (b).

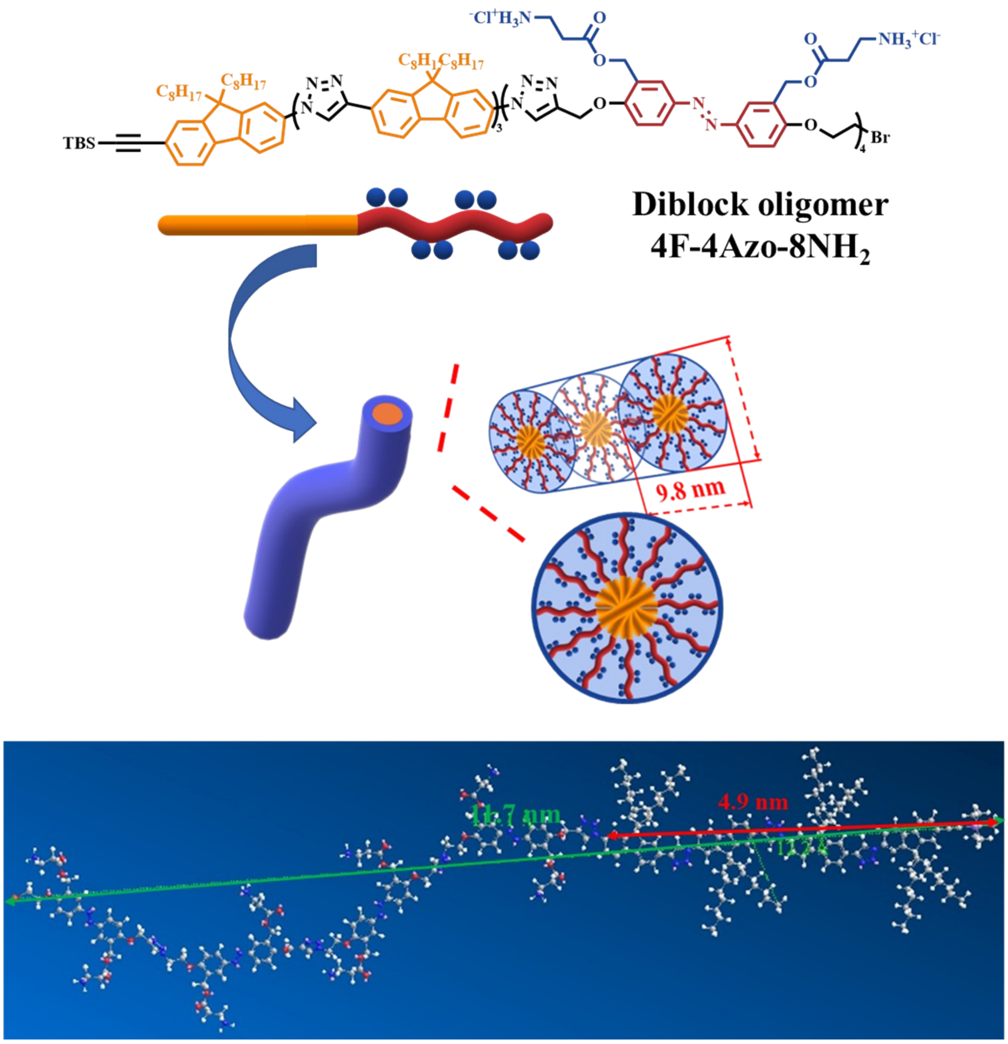

Figure S16. Schematic diagram of molecular stacking of fibers formed by the oligomer 4F-4Azo-8NH$_2$ during self-assembly in mixed solution THF/water/HCl (Top). The simulative molecular configuration of 4Azo-4F-8NH$_2$ calculated with Materials Studio software (MS). The molecular length of 4Azo-4F-8NH$_2$ is 11.7 nm, of which azobenzene block with amino side-chain groups is about 6.8 nm, and fluorene block is 4.9 nm (below).

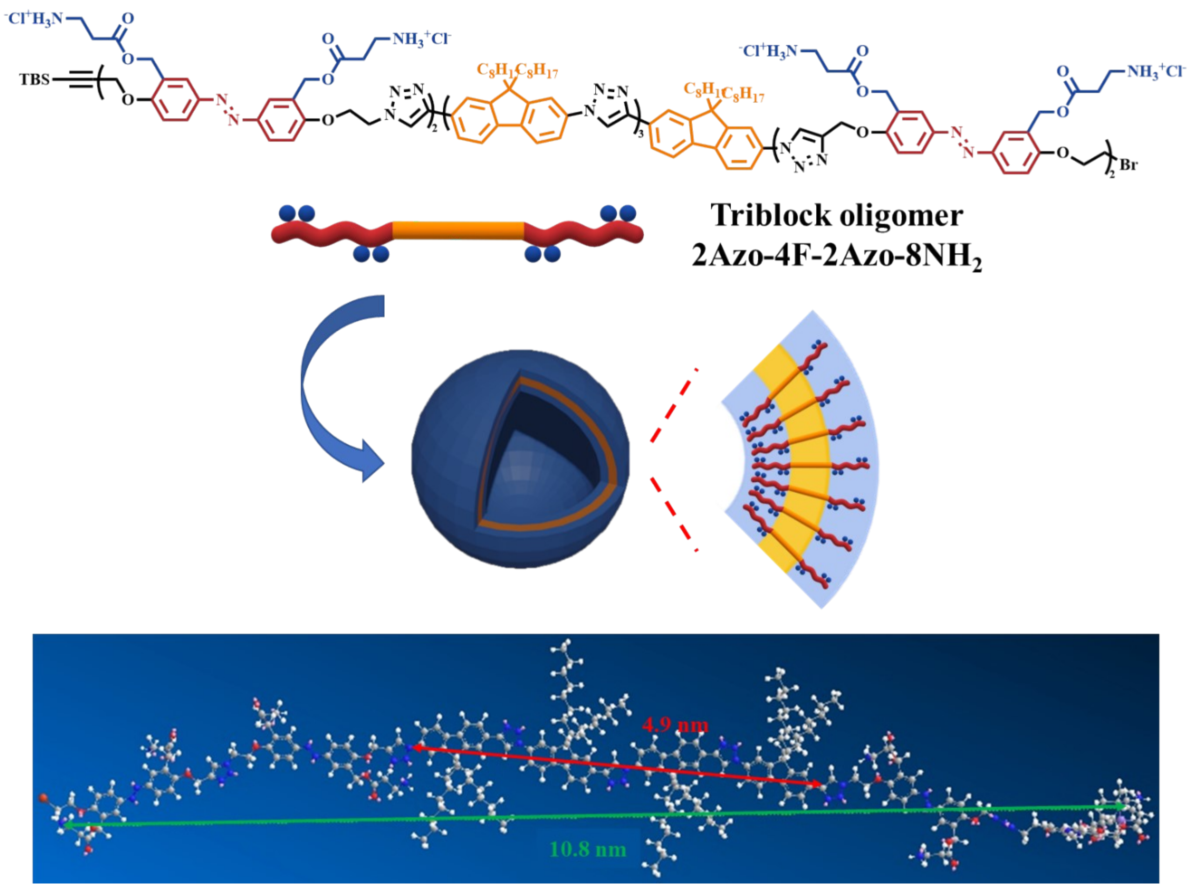

Figure S17. Schematic diagram of molecular stacking of vesicles formed by the oligomer 2Azo-4F-2Azo-8NH$_2$ during self-assembly in mixed solution THF/water/HCl (Top). The simulative molecular configuration of 2Azo-4F-2Azo-8NH$_2$ (b) calculated with Materials Studio software (MS). The molecular length of 2Azo-4F-2Azo-8NH$_2$ is 10.8 nm, of which two azobenzene blocks with amino side-chain groups is 2.95 nm respectively, and fluorene block is 4.9 nm (below).

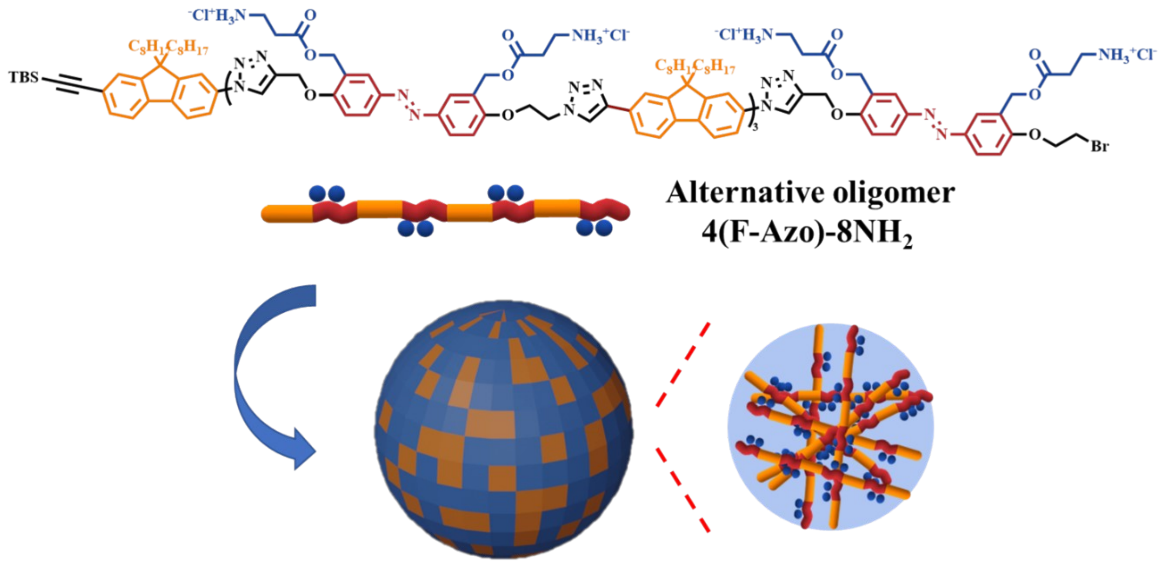

Figure S18. Schematic diagram of molecular stacking of micellar complex formed by the oligomer 4(F-Azo)-8NH_2 during self-assembly in mixed solution THF/water/HCl

References

[S1] Min Liu, Xianheng Shi, Lishan Li, Jiandong Zhang, Zhihao Huang, Wei Zhang,Nianchen Zhou,* Zhengbiao Zhang,* and Xiulin Zhu, Synthesis of Discrete Conjugated Fluorene-Azo Oligomers for the Investigation of Azobenzene Position-Dependent Physical Properties and Photoresponsive Behavior, *Macromol. Chem. Phys.,* **2021**, 2100092.

[S2] An Hu, Xianheng Shi, Lishan Li,* Wei Zhang, Zhengbiao Zhang,* Nianchen Zhou,*and Xiulin Zhu, A Consequence of Dispersity on the Self-Assembly of Amphiphilic Homopolymers Containing Main-Chain Azobenzene, *Macromol. Chem. Phys.,* **2021**, 2100202.
